# Polyphenol profiling and multi-target antioxidant, anti-inflammatory and antidiabetic activity of *Sorbus commixta* Hedl. leaves

**DOI:** 10.1038/s41598-026-57556-5

**Published:** 2026-06-13

**Authors:** Magdalena Rutkowska, Joanna Kolodziejczyk-Czepas, Oleksandra Liudvytska, Piotr Michel, Monika A. Olszewska

**Affiliations:** 1 Department of Pharmacognosy, Faculty of Pharmacy, Medical University of Lodz, 1 Muszynskiego St, Lodz, 90-151 Poland; 2 Department of General Biochemistry, Faculty of Biology and Environmental Protection, University of Lodz, Pomorska 141/143, Lodz, 90-236 Poland

**Keywords:** *Sorbus commixta*, Phenolic profile, Antioxidant, Anti-inflammatory, Antidiabetic, Antiglycation, Biochemistry, Biotechnology, Chemical biology, Drug discovery, Plant sciences

## Abstract

*Sorbus commixta*, a plant from the Rosaceae family, is native to Asia and cultivated in Europe and North America. Here, we evaluated the potential of *S. commixta* leaves as a source of biologically active compounds, including rare flavanones. The methanol-water extract (ME) and its bioactive polyphenol-enriched fractions were assessed for phytochemical composition, biological activity, and composition-activity relationships. A total of 78 compounds were identified by UHPLC-PDA-ESI-MS/MS, with a content reaching 589 mg/g of dry weight. Among the tested samples, ME and the *n*-butanol fraction (BF) exhibited the strongest biological effects. Both significantly protected human plasma components against peroxynitrite-induced oxidative and nitrative stress (ex vivo model) and inhibited the release of pro-inflammatory cytokines (IL-1β, IL-2, TNF-α) from stimulated human peripheral blood mononuclear cells in vitro. The BF fraction also demonstrated pronounced antidiabetic activity, showing approximately 17- to 26-fold stronger effects than acarbose and aminoguanidine in inhibiting α-glucosidase and the formation of advanced glycation end products in vitro. Statistical analysis revealed significant correlation of the observed effects with polymeric proanthocyanidins, flavonol diglycosides, caffeoylquinic acids, and monoglycosides of flavonols and flavanones. These findings support further in vivo investigation of *S. commixta* leaf extracts and highlight the need for their standardised quality control strategies.

**Supplementary Information:**

The online version contains supplementary material available at https://doi.org/10.1038/s41598-026-57556-5.

## Introduction

 Plants naturally produce a wide range of chemicals for their growth and development, including biologically active substances that have the potential to prevent or treat human diseases. However, only particular species can accumulate significant amounts of specialised metabolites with beneficial medicinal properties. Consequently, these taxons become valuable sources of pharmaceuticals for commercial use^1^.

The prior research has shown that many species from the genus *Sorbus* L. (Rosaceae) are rich sources of various classes of bioactive phenylpropanoids, including proanthocyanidins, caffeoylquinic acids, and flavonoids^2–8^. Among these, *Sorbus commixta* Hedl. (syn.: *S. wilfordii* Koehne), also known as Japanese rowan, stands out as one of the most promising species in this regard. Native to the Asian continent, this small- to medium-sized deciduous tree is used in traditional phytotherapy to treat conditions such as atherosclerosis, diabetes, asthma, or rheumatism^8,9^. The bark of *S. commixta* is particularly favoured for medicinal use, whether in mixed formulations or as a standalone remedy^9^. Thanks to the ease of cultivation beyond its natural habitat, the Japanese rowan has been introduced worldwide, gaining special popularity in Europe and North America^8^.

Even though *S. commixta* bark is the best-recognised plant material, with documented antioxidant, anti-diabetic, anti-inflammatory, anti-atherogenic, neuroprotective, and hepatoprotective benefits^8,10,11^, the harvesting of bark from trees or shrubs is restricted and requires breaks to conserve plants^12^. Therefore, the commercial availability of Japanese rowan bark is limited. In contrast, leaves of deciduous trees are cost-effective plant materials readily available throughout the whole growing season. In addition, in the case of *Sorbus* species, leaves tend to accumulate elevated amounts of phenylpropanoids^2,4,5,8^. For instance, previous studies revealed the total phenolic content in the *S. commixta* leaves reaching 80–125 mg GAE (gallic acid equivalents) per g of dry leaves, which positioned this material at the top of tested samples (leaves, flowers, fruits, bark) from over thirty *Sorbus* species and defined it as one of the most efficient sources of polyphenols in general^3,7,8,13,14^. However, while the total polyphenolic content was exceptionally promising for this plant material, the scientific data on its chemical composition, the structures of individual constituents, and biological activity are limited to single compounds and preliminary tests of the antioxidant capacity in simple, non-cellular DPPH and FRAP in vitro assays^4,5^.

The present study aimed to thoroughly characterise the composition of the polyphenolic fraction of *S. commixta* leaves, evaluate their antioxidant, anti-inflammatory, and antidiabetic potential in vitro, as well as identify compounds or phenylpropanoid classes that primarily contribute to these effects. To achieve this task, we compared the phytochemical and biological profiles of the crude leaf extract of *S. commixta* and its polyphenol-enriched fractions obtained through fractionated extraction. Fractionation enabled the identification of a broader range of compounds, allowing more precise evaluation of structure-activity relationships and the selection of the most active products possible with sufficient extraction yield. The activity directions chosen for this study included those most frequently reported by the phytotherapeutic tradition for various *Sorbus* materials, including *S. commixta* bark, as well as effects known for *Sorbus*-typical polyphenols. The qualitative UHPLC-PDA-ESI-MS/MS and quantitative HPLC-PDA assays were used in the phytochemical part of the study, while the activities were tested using complementary cellular tests (on peripheral blood mononuclear cells, PBMCs), cell-free in vitro assays, and ex vivo human plasma models related to oxidative/nitrative stress, inflammation, and diabetes. The composition-activity relationships were tested in the correlation studies, and the primary active markers were selected for the extracts. Prior to the cellular tests, the extracts were evaluated for cytotoxicity and confirmed to be biocompatible over the tested concentration range.

## Materials and methods

### Plant material and extracts preparation

Leaves of *Sorbus commixta* Hedl. (syn.: *S. wilfordii* Koehne, *Sorbus commixta* var. *rufoferruginea* Shirai ex C.K.Schneid.; Japanese rowan) were collected in the Arboretum, Forestry Experimental Station of Warsaw University of Life Sciences (SGGW) in Rogow (Poland) (51°49′N, 19°53′E), authenticated by the Head of the Arboretum, MSc Eng. Piotr Banaszczak, and harvested with the approval of the institution and in accordance with its guidelines. No endangered or protected plant species were involved in the study.

**Fig. 1:**
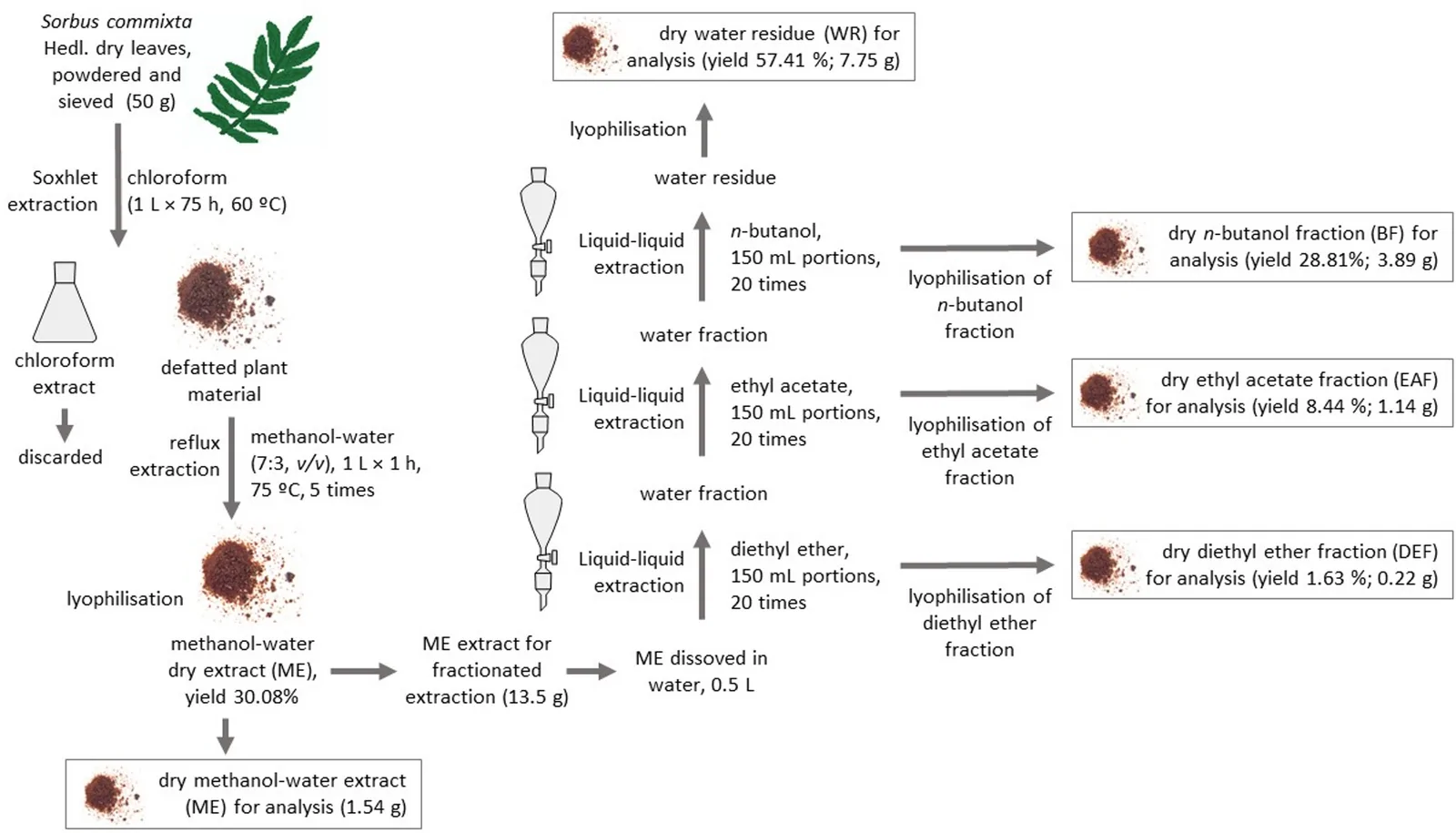
The procedure of extract preparation and liquid-liquid fractionation (figure prepared by the author with the use of PowerPoint and Paint programmes, and photos taken by the author).

Plant material was air-dried under normal conditions, powdered, and sieved (*φ* = 0.315 mm). The voucher specimen (KFG/HB/15006-SCOM/L) was deposited in the Medicinal Plant Garden. The plant name was checked and revised according to the World Flora Online Plant List^15^. The dry leaf sample underwent defatting via chloroform pre-extraction using a Soxhlet apparatus. The resulting defatted material was subsequently extracted with a methanol-water mixture (7:3, *v/v*), followed by sequential liquid–liquid partitioning with solvents of increasing polarity, as described in detail in Fig. 1. Consequently, five dry extracts/fractions were prepared for further analysis, i.e., the methanol-water extract (ME), diethyl ether fraction (DEF), ethyl acetate fraction (EAF), *n*-butanol fraction (BF), and the water residue (WR).

The selection of extraction conditions was based on their suitability for efficient recovery of structurally diverse polyphenols from *S. commixta* leaves. The methanol-water systems provide optimal polarity for simultaneous extraction of both less polar compounds (e.g., flavonoid aglycones) and more polar constituents, including phenolic acids and glycosylated flavonoids^2,16^. Prior removal of lipophilic matrix components (e.g., chlorophylls, waxes) improves extraction selectivity and reduces analytical interferences; the chloroform fraction was therefore discarded after confirming the absence of detectable polyphenols^17^. The solvent composition was optimised by testing different alcohol-water systems, with the 7:3 (*v/v*) mixture yielding the highest recovery of polyphenols.

The crude methanol-water extract was subsequently subjected to liquid-liquid fractionation using solvents of increasing polarity to yield fractions enriched in compounds with similar physicochemical properties. This step reduced sample complexity, improved chromatographic resolution, and enhanced the detection of minor constituents, enabling more reliable identification of a broader range of compounds. Furthermore, it facilitated the assessment of structure-activity relationships by linking biological activity to specific fractions^2,4,16^.

## Phytochemical profiling

The qualitative studies were performed using a UHPLC-PDA-ESI-MS/MS technique and a UHPLC-3000 RS chromatographic system from Dionex (Dreieich, Germany), coupled with an AmaZon SL ion trap mass spectrometer featuring an ESI interface from Bruker Daltonik (Bremen, Germany). Separation of samples dissolved in 70% aqueous methanol (concentrations 5 mg/mL) was achieved using a Kinetex XB-C18 column (1.7 μm, 150 mm × 2.1 mm i.d.; Phenomenex, Torrance, CA, USA) and the previously optimised analytical procedure^2^. Analyses were carried out at 25 °C with a flow rate of 0.3 mL/min using water (A) and acetonitrile (B) (both containing 0.1% formic acid, *v/v*) as mobile phases. The elution gradient progressed from 6% to 26% B (0–45 min), then to 95% B (45–55 min), held until 63 min, and subsequently returned to initial conditions by 70 min, followed by re-equilibration to 80 min. MS data were recorded over an m/z range of 100–2200. ESI parameters included a drying gas (9 L/min, 300 °C), nebuliser pressure of 40 psi, and capillary voltage of 4.5 kV.

Quantitative HPLC-PDA analyses were conducted using a Waters 600E Multisolvent Delivery System (Waters, Milford, MA, USA), following the previously described method^4^. Samples of the extracts and obtained fractions were dissolved in 70% aqueous methanol to achieve concentrations of 1–5 mg/mL, depending on the fraction, and subsequently injected into the HPLC system. The mobile phase consisted of solvent A (0.5% orthophosphoric acid in water, *w/v*) and solvent B (acetonitrile). Separation was achieved using a linear gradient starting at 5% B (0–1 min), increased to 30% B over 1–16 min, then to 50% B within 1 min (16–17 min), held at 50% B until 19 min, followed by reversion to initial conditions (5% B) over 19–20 min and equilibration until 25 min. The flow rate was maintained at 1.4 mL/min, and the column temperature was set to 30 °C. Detection and classification of phenolic compounds were based on their UV–Vis spectral characteristics and retention times, and the peak purity assessment was performed using an automated spectral matching system (Waters Empower 2 PDA) by comparison with the reference standards.

Quantification was carried out using an external calibration with fourteen reference standards (Sigma-Aldrich, Seelze, Germany/St. Louis, MO, USA), including caffeic acid (CFA), chlorogenic acid (CHA), cynarin (CYN), *p*-coumaric acid (pCA), protocatechuic acid (PCA), *p*-hydroxybenzoic acid (pHBA), (-)-epicatechin (EC), rutin (RT), isoquercitrin (IQ), hyperoside (HY), quercetin 3-*O*-sophoroside (SQ), naringin (NG), quercetin (QU), eriodyctiol (ER). Calibration curves were constructed for each standard over an appropriate concentration range, and all curves exhibited good linearity (R² ≥ 0.998). Method precision, evaluated as intra- and inter-day repeatability, was within 2–5% relative standard deviation (RSD). Method accuracy, assessed via recovery experiments, amounted to 95–105%. Limits of detection (LOD) and quantification (LOQ) were determined based on signal-to-noise ratios of 3 and 10, respectively, and fell within 0.2–1.0 µg/mL and 0.5–3.0 µg/mL, depending on the compound. For the analytes lacking authentic standards, semi-quantitative estimation was performed using calibration curves of structurally related compounds within the same phenolic subclass, a strategy justified by their similar chromophoric units and comparable molar absorptivity (e.g., neochlorogenic acid was quantified as chlorogenic acid equivalents). The total phenolic content (TPH) was calculated as the sum of individual analytes and expressed as mg per g of the dry extract/fraction weight (mg/g dw).

The total phenolic content (TPC) was also determined using spectrophotometric, Folin-Ciocalteu (FC) assay, with the procedure reported by Olszewska and Michel^18^. The methanol-water (7:3, *v/v*) solutions of the tested extract and fractions (100–250 µg/mL) were mixed with the FC reagent (Sigma-Aldrich, Seelze, Germany/St. Louis, MO, USA), water, and Na_2_CO_3_·10 H_2_O (1 M in water) using the volume ratio of 2:1:9:8. After 2 h of incubation in the dark, the absorbance was measured at 760 nm using a SPECTROStarNano microplate reader (BMG Labtech, Ortenberg, Germany) against blank samples without FC reagent. Results were expressed as gallic acid equivalents (GAE) (based on the gallic acid calibration curve) per dry weight of extract/fraction (mg GAE/g dw).

The total proanthocyanidin content (TPA) was determined by the *n*-butanol-HCl method as described by Olszewska et al.^4^. The methanol-water (7:3, *v/v*) solutions of the tested extract and fractions (0.5–2.0 mg/mL) were mixed with n-BuOH-35% HCl (10:3, *v/v*) and NH_4_Fe(SO_4_)_2_·12 H_2_O (2% in 2 M HCl) using the volume ratio of 5:30:1. After 45 min incubation in the water bath (95 °C), the absorbance was measured at 550 nm using a SPECTROStarNano microplate reader (BMG Labtech, Ortenberg, Germany) against blank, i.e., unheated samples. Results were expressed as cyanidin chloride equivalents (CyE) (based on the cyanidin chloride calibration curve) per dry weight of extract/fraction (mg CyE/g dw).

## Antidiabetic activity testing

The inhibitory effects on enzymes involved in carbohydrate digestion, i.e., α-glucosidase and α-amylase, and the influence on non-enzymatic protein glycation (the formation of advanced glycation end-products, AGEs) were tested using spectrophotometric and fluorimetric methods as described by Kicel et al.^19^, and performed on SPECTROStar Nano microplate reader (BMG Labtech, Ortenberg, Germany), UV-1601 Rayleigh spectrophotometer (Beijing, China), and multilabel counter Victor 1420 (Perkin Elmer Life and Analytical Sciences, Shelton, CT, USA).

For α-glucosidase inhibition, the assay mixture consisted of α-glucosidase (0.43 U/mL in 0.1 M phosphate buffer, pH 6.8) and the tested samples (in phosphate buffer) at various concentrations. They were incubated for 15 min at 37 °C, followed by the addition of substrate solution, i.e., *p*-nitrophenyl-α-D-glucopyranoside (0.2 mg/mL in phosphate buffer), and further incubation for 15 min, finally ended with the use of Na_2_CO_3_ (0.2 M in phosphate buffer). The reagents were used in the volume ratio of 1:3:1:1. The amount of released *p*-nitrophenol was determined spectrophotometrically at 405 nm. The inhibitory activity was expressed as IC₅₀ values, calculated from concentration-response curves constructed using 4–5 concentration points (concentration range amounted to 5–50 µg/mL or 50–200 µg/mL, depending on the extract/fraction). Acarbose (AR) was used as a reference inhibitor with IC_50_ = 173.3 µg/mL.

For α-amylase inhibition, the assay mixture consisted of α-amylase solution (2.6 U/mL in 0.02 M phosphate buffer, pH 6.6; 400 µL) and the tested samples (in phosphate buffer) at various concentrations. They were incubated for 15 min at room temperature, then the reaction was initiated by the addition of 0.8% starch solution in water, followed by a further 15 min incubation at room temperature. The enzymatic reaction was terminated by adding the dinitrosalicylic acid reagent (the mixture of 3,5- dinitrosalicylic acid 35 mM, C_4_H_4_O_6_KNa·4H_2_O 1 M, and NaOH 0.4 M in water) and phosphate buffer. The final working mixtures (volume ratio of reagents 4:8:4:3:5) were heated at 100 °C for 10 min, cooled, and the absorbances were measured at 540 nm. Inhibitory activity was expressed as IC₅₀ values calculated from concentration-response curves based on 4–5 concentration points (concentration range reached 30–150 µg/mL, 100–600 µg/mL, or 300–1500 µg/mL, depending on the extract/fraction). Acarbose (AR) was used as a positive control with IC_50_ = 9.8 µg/mL.

For inhibition of AGEs formation, the working solution consisted of bovine serum albumin (BSA, 10 mg/mL in 0.1 M phosphate buffer, pH 7.4, with addition of 0.01% sodium azide to prevent microbial growth), fructose (90 mg/mL in phosphate buffer), and the tested samples (in phosphate buffer) at various concentrations (volume ratio of reagents 1:1:1). The mixtures were incubated in the dark at 37 °C for six days, followed by the fluorescence measurement with excitation and emission wavelengths set at 355 nm and 420 nm, respectively. The antiglycation activity was expressed as IC₅₀ values calculated from concentration-response curves constructed using 4–5 concentration points (concentration range reached 2.5–15 µg/mL or 10–20 µg/mL, depending on the extract/fraction). Aminoguanidine (AG) was used as a positive control with IC_50_ = 79.1 µg/mL.

To facilitate comparison of inhibitory potency between extracts and reference compounds across assays, the IC₅₀ values were recalculated as equivalents of the corresponding standards (µmol of standard per mg of extract/fraction dw), providing a normalised measure of biological effectiveness and ensuring consistency of activity parameters obtained in different assays. Hence, the inhibitory activity against α-glucosidase was calculated based on Eq. 1, against α-amylase – based on Eq. 2, and antiglycation activity – based on Eq. 3.

1 $$\:\mathrm{Activity\:}\alpha\mathrm{-glucosidase}\mathrm{\:(}\frac{\mu\mathrm{mol\:AR}}{\mathrm{mg\:of\:sample}}\mathrm{)=}\frac{\mathrm{173.3\:}\left(\mathrm{AR\:IC}\mathrm{50}\mathrm{;}\frac{\mu\mathrm{g}}{\mathrm{mL}}\right)\times\mathrm{\:1000}}{\mathrm{646.6\:}\left(\mathrm{AR\:molecular\:weight;}\frac{\mathrm{g}}{\mathrm{mol}}\right)\times\mathrm{\:IC}\mathrm{50}\mathrm{\:(tested\:sample;\:}\frac{\mu\mathrm{g}}{\mathrm{mL}}\mathrm{)}}$$

2 $$\:\mathrm{Activity\:}\alpha\mathrm{-amylase}\left(\frac{\mu{\mathrm{mol}\:AR}}{\mathrm{mg\:of\:sample}}\right)=\frac{\mathrm{9.8\:}\left(\mathrm{AR\:IC}\mathrm{50}\mathrm{;}\frac{\mu\mathrm{g}}{\mathrm{mL}}\right)\times \mathrm{1000}}{\mathrm{646.6\:}\left(\mathrm{AR\:molecular\:weight;}\frac{\mathrm{g}}{\mathrm{mol}}\right)\times \mathrm{\:IC}\mathrm{50}\mathrm{\:(tested\:sample;\:}\frac{\mu \mathrm{g}}{\mathrm{mL}}\mathrm{)}}$$

3 $$\:\mathrm{Antiglycation\:activity}\mathrm{\:(}\frac{\mu\mathrm{mol\:AG}}{\mathrm{mg\:of\:sample}}\mathrm{)=}\frac{\mathrm{79.1\:}\left(\mathrm{AG\:IC}\mathrm{50};\frac{\mu\mathrm{g}}{\mathrm{mL}}\right)\times \mathrm{1000}}{\mathrm{74.1\:}\left(\mathrm{AG\:molecular\:weight;}\frac{\mathrm{g}}{\mathrm{mol}}\right)\times \mathrm{IC}\mathrm{50}\mathrm{\:(tested\:sample;\:}\frac{\mu\mathrm{g}}{\mathrm{mL}}\mathrm{)}}$$

## Antioxidant activity testing

The influence of the extracts on human plasma components and the non‑enzymatic antioxidant capacity of plasma (NEAC) was evaluated using fully anonymised buffy coats purchased at the Regional Centre of Blood Donation and Blood Treatment in Lodz, Poland. Both the blood collection at the Centre of Blood Donation and Blood Treatment (Lodz, Poland) and the sale of buffy coats were conducted in accordance with applicable legal and institutional regulations, including the Regulation (EU) 2016/679 of the European Parliament and of the Council of 27 April 2016 concerning the protection of natural persons with regard to the processing of personal data (GDPR). Since the material was purchased, no recruitment of volunteers, collection of personal data, or direct interaction with blood donors was involved in this study. The experiments were approved by the Committee for Bioethics of Scientific Research of the University of Lodz (3–4/KBBN-UŁ/II/2020-21).

Plasma samples were obtained by differential centrifugation of blood as previously described^20^, incubated with the tested extracts (1–50 µg/mL) at 37 °C for 15 min, and then treated with 100–150 µM ONOO^−^. Notably, in experiments involving extracts alone (without ONOO^−^), no pro-oxidative effects were observed. Each parameter measured in plasma or peripheral mononuclear blood cells (PBMCs) was assessed using samples obtained from several independent donors; therefore, the values of “*n*” refer to the number of independent experiments. The concentration range for tested extracts/fractions was selected to enable evaluation of the biological activity under varying experimental conditions, including both low and high exposure levels, and to allow comparison with previously reported studies on polyphenol-rich plant materials^21–23^.

The antioxidant activity parameters were measured using immunoenzymatic and spectrophotometric methods, and presented as relative percentages with the values obtained for ONOO⁻‑treated control plasma (without extracts) assumed as 100%. Trolox (TX) was used as a positive control.

The level of 3-nitrotyrosine in plasma proteins was assessed using a competitive enzyme-linked immunosorbent assay (ELISA). Microtiter plates were pre‑coated with a nitrated protein antigen (human fibrinogen). Samples or standards were mixed with an anti‑3‑nitrotyrosine antibody and added to the wells, allowing competition between plate‑bound and sample-derived antigen. After incubation, unbound proteins were removed by washing, and the plate was blocked with 1% defatted milk solution (in 0.1 M tris/HCl buffer, pH 9), followed by the addition of a biotinylated secondary antibody and subsequently the streptavidin-HRP conjugate. The enzymatic reaction was developed using *O*-phenylenediamine dihydrochloride (OPD). The concentration of 3‑nitrotyrosine (3-NT) ‑containing proteins in plasma was determined using a standard curve and expressed as nmol of 3‑NT‑containing fibrinogen equivalents per mg of plasma protein^2,20^.

Lipid hydroperoxides (LOOHs) were quantified using the ferric-xylenol orange (FOX-1) assay. The xylenol orange assay allows for the quantification of lipid hydroperoxides (LOOHs) based on their ability to oxidise ferrous ions (Fe²⁺) to ferric ions (Fe³⁺) under acidic conditions. Subsequently, Fe³⁺ ions react with xylenol orange and form purple-coloured complexes which can be detected spectrophotometrically. The assay was carried out according to the previously described protocol^24^. The FOX‑1 working solution consisted of 125 µM xylenol orange and 100 mM sorbitol dissolved in 25 mM sulfuric acid. Immediately before use, ammonium ferrous sulphate hexahydrate (Mohr’s salt) was added to achieve a final concentration of 250 µM. For the FOX‑1 assay, plasma samples were combined with the reagent at a 1:9 volume ratio and incubated for 30 min at 25 °C in the dark. Absorbance was then recorded at 560 nm. LOOH levels were quantified based on a hydrogen peroxide calibration curve and expressed as nmol per milligram of plasma proteins.

End products of lipid peroxidation were determined using the thiobarbituric acid (TBA). The assay was based on the ability of the lipid peroxidation products (mainly MDA) to react with TBA and yield a pink chromogenic adduct, detected spectrophotometrically (at λ = 532 nm). The experiments were carried out according to the previously described protocol^25^. Briefly, each sample was mixed with an equal volume of 20% (*v/v*) trichloroacetic acid prepared in 0.6 M HCl, followed by centrifugation at 1,200 × g for 15 min. The resulting clear supernatant was then collected and combined with 0.12 M TBA in a volume ratio of 1:1. Subsequently, the samples were incubated at 100 °C for 10 min, cooled immediately in an ice bath, and centrifuged to obtain clear supernatants. The TBA-reactive substance level was expressed as µmol/mL of plasma.

The non-enzymatic antioxidant capacity of plasma (NEAC) was determined based on its ability to reduce ferric (Fe³⁺) ions to ferrous (Fe²⁺) ions (the FRAP assay). During this test, antioxidants present in the assayed sample reduce the Fe³, and the generated Fe²⁺ ions form a blue complex with 2,4,6‑tripyridyl‑s‑triazine (TPTZ). Plasma samples were first diluted 4-fold and subsequently combined with the reagent mixture using a volume ratio of 1:10:1:1 for plasma, acetate buffer (300 mM, pH 3.6), TPTZ solution (10 mM prepared in 0.04 M HCl), and FeCl₃ (20 mM), respectively. The absorbance was measured at 593 nm, and the reducing ability was expressed as Fe^2+^ equivalents [millimoles/L]^24^.

## Anti-inflammatory activity testing

The effect on the release of pro-inflammatory cytokines was tested in the PBMCs model as described earlier^20^. The cells (1.5 × 10^6^ cells/mL) were isolated from fresh buffy coats (material described in the “Antioxidant activity testing” section), suspended in RPMI 1640 medium (BioWest, Nuaillé, France), and then seeded into 96‑well microplates at a density of 3.75 × 10⁵ cells per well. PBMCs were subsequently pre-incubated for 1 h with the *S. commixta* extracts (final concentrations of: 1–50 µg/mL) under standard CO₂-incubator conditions (5% CO₂, 95% humidity). After this pre-incubation period, the cells were treated with concanavalin A (Con A) at a final concentration of 10 µg/mL and maintained in culture for an additional 24 h. Following the incubation, the plates were centrifuged to collect supernatants from the culture medium. The levels of cytokines (IL-1β, IL-2, IL-6, and TNF-α) in the collected cell medium were determined using commercial ELISA kits (OptEIA ELISA Sets, Becton Dickinson, Franklin Lakes, New Jersey), following the manufacturer’s protocols. Indomethacin (IND), diclofenac (DIC), and dexamethasone (DEX) were used as positive controls. The anti-inflammatory potential of the tested plant extracts was determined by comparing the levels of pro-inflammatory cytokines in cells pre-treated with the extracts and subsequently stimulated with Con A to the Con A-stimulated cells in the absence of the *S. commixta* extracts (stimulated control). To establish the baseline cytokine release, additional control samples without Con A stimulation (unstimulated controls) were also prepared.

### Influence on cell viability

The cells (PBMCs, 1.5 × 10⁶ cells/mL, suspended in RPMI 1640 medium) were exposed to *S. commixta* extracts (1–50 µg/mL) and subsequently cultured for 24 h in 96‑well plates, with 3.75 × 10⁵ cells seeded per well, under standard conditions (37 °C, 5% CO₂, 95% humidity)^20^. Reference wells, serving as the 0% viability, contained PBMCs treated with 1% Triton X‑100, while control wells included cells that received no treatment. The TOX8 in vitro toxicology assay kit was used to evaluate the cytotoxicity of the extracts on PBMCs. The protocol followed the manufacturer’s guidance (Sigma-Aldrich/Merck, Darmstadt, Germany). Cell viability was determined spectrophotometrically using a microplate reader BMG Labtech SectroStarNano (λ = 600 nm, with a reference wavelength of 690 nm).

## Statistical analyses

The results were shown as means ± SD (standard deviation) or ± SE (standard error). For phytochemical profiling and antidiabetic activity testing, all samples were analysed in three independent experiments, each performed in triplicate. For anti-inflammatory tests, the number of experiments for each sample was eight to ten, while for antioxidant tests, it was four to eight experiments on plasma samples from independent donors. The statistical significance of differences between the means was evaluated using a one-way ANOVA (antidiabetic and anti-inflammatory tests) or one-way ANOVA for repeated measures (antioxidant tests), followed by the post-hoc Tukey’s (antidiabetic tests) test for multiple comparisons or post-hoc Dunnett’s test (antioxidant and anti-inflammatory tests). The Pearson correlation coefficients were used to test the relationships between variables. Satistica13.3Pl software (StatSoft Inc., Krakow, Poland) for Windows was used for all statistical analyses, with *p*-values less than 0.05 regarded as significant.

## Results

### Fractionated extraction and phytochemical profiling

**Fig. 2:**
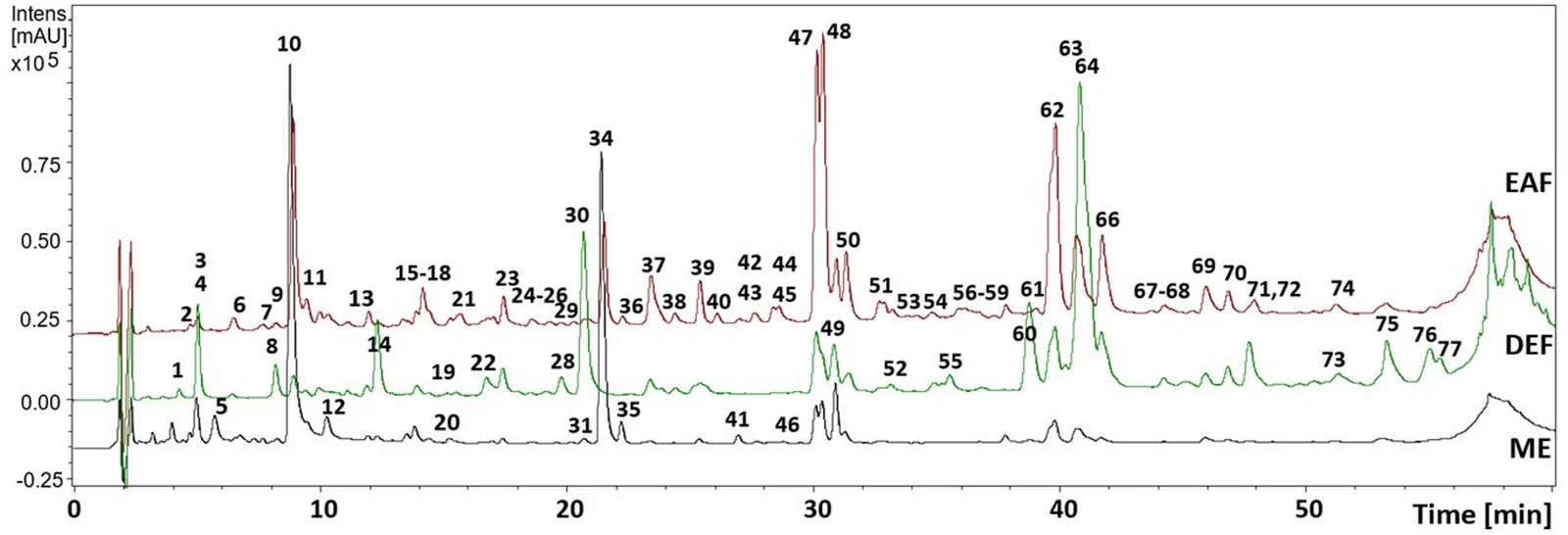
UHPLC-MS/MS chromatograms of the ME extract and DEF and EAF fractions from *S. commixta* leaves (200–450 nm). The numbers refer to detected compounds (see below). For image readability, some numbers were omitted; hence, for full identification, see Supplementary Table S1 online.

As different classes of polyphenols have been repeatedly reported as the primary constituents of *Sorbus* plants^3,7,8,13,14^, the tested *S. commixta* leaves were first extracted with the solvent previously recommended, i.e., methanol-water mixture (7:3, *v/v*). This solvent was also confirmed to provide the highest recovery of *S. commixta* polyphenols (extraction yield: 29.8%) through preliminary optimisation. Next, liquid-liquid partitioning was performed, yielding the total fractionation of 96.3% and three subfractions (DEF, EAF, BF), each enriched in different active constituents. The subfractions were subjected to LC-MS/MS analysis and structural identification (complete or tentative) of individual compounds by comparing their spectral profiles and retention times with reference standards or literature data^3,4,6,26–33^. The results allowed us to categorise the tested fractions into those accumulating low-molecular-weight polyphenols (DEF and EAF) and those enriched in medium-weight polyphenols up to high-molecular-weight proanthocyanidins (BF). All in all, 78 phenolic compounds were detected (Fig. 2), including phenolic acids (34 compounds), flavonols (14 compounds), flavan-3-ols (20 compounds), flavanones (4 compounds), and flavalignans (6 compounds).

The complete list of identified compounds is as follows: **(1)** protocatechuic acid hexoside, **(2)** caffeic acid hexoside, **(3)** protocatechuic acid (PCA), **(4)** 3-*O*-caffeoylquinic acid (NCHA, neochlorogenic acid), **(5)** 5-*O*-caffeoylquinic acid hexoside, **(6)** (epi)catechin-B-(epi)catechin, **(7)** (epi)catechin-B-(epi)catechin, **(8)** *p*-hydroxybenzoic acid (pHBA), **(9)** (epi)catechin-B-(epi)catechin, **(10)** 5-*O*-caffeoylquinic acid (chlorogenic acid, CHA), **11.** (+)-catechin, **12.** 4-*O*-caffeoylquinic acid (CCHA, cryptochlorogenic acid), **13.** caffeic acid acetyl-hexoside, **14.** caffeic acid (CFA), **15.** (epi)catechin-B-(epi)catechin, **16.** procyanidin B2, **17.** caffeoylmalic acid, **18.** cis-5-*O*-caffeoylquinic acid (cis-CHA, cis-chlorogenic acid), **19.** caffeic acid acetyl-hexoside, **20.** 5-*O*-*p*-coumaroylquinic acid (5-CQA), **21.** (–)-epicatechin (EC), **22.** caffeic acid acetyl-hexoside, **23.** 5-*O*-caffeoylshikimic acid (5-CFSA), **24.** (epi)catechin-B-(epi)catechin hexoside, **25.** caffeic acid acetyl-hexoside, **26.** caffeic acid hexoside derivative, **27.** quercetin *O*-rhamnosyl-hexosyl-hexoside, **28.** vanillic acid, **29.** (epi)catechin-B-(epi)catechin-B-(epi)catechin, **30.** *p*-coumaric acid (pCA), **31.** quercetin *O*-dihexoside, **32.** (epi)catechin-B-(epi)catechin hexoside, **33.** (epi)catechin-B-(epi)catechin-B-(epi)catechin, **34.** quercetin 3-*O*-β-sophoroside (SQ), **35.** quercetin *O*-dihexoside (diHQ), **36.** (epi)catechin-B-(epi)catechin, **37.** (epi)catechin-B-(epi)catechin hexoside, **38.** 5-*O*-feruloylquinic acid, **39.** caffeic acid hexoside derivative, **40.** (epi)catechin-B-(epi)catechin hexoside, **41.** kaempferol *O*-dihexoside (diHK), **42.** (epi)catechin-B-(epi)catechin hexoside, **43.** kaempferol *O*-dihexoside, **44.** (epi)catechin-B-(epi)catechin hexoside, **45.** (epi)catechin-B-(epi)catechin hexoside, **46.** quercetin *O*-hexosyl-pentoside, **47.** eriodictyol *O*-hexoside 1 (EH1), **48.** eriodictyol *O*-hexoside 2 (EH2), **49.** Quercetin 3-*O*-β-D-(6′′-*O*-α-L-rhamnosyl)-glucoside (RT, rutin), **50.** quercetin 3-*O*-β-D-galactoside (HY, hyperoside), **51.** quercetin 3-*O*-β-D-glucoside (IQ, isoquercitrin), **52.** caffeic acid hexoside derivative, **53.** kaempferol *O*-hexoside, **54.** (epi)catechin derivative, **55.** cinchonain I isomer, **56.** (epi)catechin-B-(epi)catechin-B-(epi)catechin hexoside, **57.** (epi)catechin-B-(epi)catechin-B-(epi)catechin hexoside, **58.** kaempferol *O*-hexoside, **59.** kaempferol *O*-rhamnosyl-hexoside, **60.** ferulic acid isomer (FAI), **61.** caffeic acid derivative, **62.** naringenin *O*-hexoside (NH), **63.** 3,5-*O*-dicaffeoylquinic acid (3,5-diCFQA), **64.** Cinchonain I isomer, **65.** caffeic acid acetyl-hexoside derivative, **66.** 1,5-*O*-dicaffeoylquinic acid (1,5-diCFQA), **67.** caffeic acid acetyl-hexoside derivative, **68.** caffeic acid acetyl-hexoside derivative, **69.** 4,5-*O*-dicaffeoylquinic acid (4,5-diCFQA), **70.** caffeic acid acetyl-hexoside derivative, **71.** cinchonain I isomer, **72.** caffeic acid acetyl-hexoside derivative, **73.** cinchonain I isomer derivative, **74.** caffeic acid derivative, **75.** cinchonain I isomer, **76.** cinchonain I isomer derivative, **77.** eriodictyol (ER), **78.** quercetin (QU). For detailed identification, see Supplementary Table S1 online.

The quantitative HPLC-PDA analysis confirmed that both the total phenolic content (TPH) and the levels of individual compounds in the extracts were high (Table 1). The TPH levels, calculated as a sum of particular peaks based on specific calibration curves, ranged from 233.5 mg/g dw (ME) to 380.18–597.9 mg/g dw (BF < DEF < EAF). Similar values were reached using the Folin-Ciocalteau method (TPC values) for all extracts, except for the DEF, where an overestimation of TPC occurred due to the richness in phenolic acids with strong reducing properties. Phenolic acids were the most abundant group of phenolic compounds in *S. commixta* leaves (Table 1). The total content of phenolic acids (TPHA) ranged from 171.4 to 404.0 mg/g dw (59–75% of TPH), with the predominant caffeoylquinic acids (TCHA up to 276.2 mg/g dw in EAF). The total flavonoid content (TFL, the sum of flavonols, flavanones, flavanols, and flavalignans) was also in the elevated range of 62.13–193.9 mg/g dw (25–35% of TPH), with the predominant flavanones (up to 148.7 mg/g dw in EAF) (Table 1). Regarding proanthocyanidins, the highest content was found in the BF fraction (TPA 186.3 mg CyE/g dw, spectrophotometric test). They were likely polymers, as they could not be detected by RP-LC-MS/MS and RP-HPLC-PDA analyses^26^.

**Table 1:** Quantitative composition of *S. commixta* leaf extract/fractions (mg/g dw).

Compounds	ME	DEF	EAF	BF	WR
PCA	n.d.	12.8±0.17 ^A^	n.d.	n.d.	n.d.
pHBA	n.d.	1.74±0.03 ^A^	n.d.	n.d.	n.d.
FAI	n.d.	29.05±0.05 ^A^	n.d.	n.d.	n.d.
CFA	n.d.	33.92±0.40 ^A^	n.d.	n.d.	n.d.
NCHA	12.04±0.22 ^B^	0.67±0.01 ^E^	5.51±0.07 ^D^	20.58±0.17 ^A^	9.69±0.20 ^C^
CHA	110.75±1.59 ^C^	33.90±0.37 ^E^	140.76±1.11 ^B^	177.20±0.28 ^A^	84.59±2.13 ^D^
CCHA	2.87±0.04 ^E^	8.99±0.09 ^A^	7.08±0.16 ^B^	4.41±0.04 ^C^	3.48±0.09 ^D^
cis-CHA	19.09±0.27 ^C^	n.d.	52.38±0.39 ^A^	29.76±0.13 ^B^	10.24±0.21 ^D^
3,5-diCFQA	3.42±0.03 ^B^	26.58±0.24 ^A^	28.12±0.21 ^A^	n.d.	n.d.
1,5-diCFQA	2.57±0.02 ^C^	20.35±0.02 ^B^	29.02±0.14 ^A^	n.d.	n.d.
4,5-diCFQA	1.79±0.01 ^C^	8.79±0.07 ^B^	13.30±0.06 ^A^	n.d.	n.d.
5-CFSA	2.83±0.03 ^B^	20.2±0.21 ^A^	20.50±0.14 ^A^	n.d.	n.d.
pCA	n.d.	45.11±0.08 ^A^	n.d.	n.d.	n.d.
5-CQA	2.73±0.00 ^C^	n.d.	12.35±0.09 ^A^	4.81±0.06 ^B^	0.66±0.01 ^D^
SQ	19.65±0.25 ^B^	n.d.	2.23±0.06 ^C^	71.36±0.45 ^A^	1.34±0.02 ^D^
RT	3.84±0.03 ^B^	n.d.	3.00±0.09 ^C^	13.51±0.17 ^A^	n.d.
diHK	0.98±0.01 ^B^	n.d.	< LOQ ^C^	3.58±0.05 ^A^	n.d.
diHQ	5.72±0.09 ^B^	n.d.	< LOQ ^C^	18.75±0.22 ^A^	n.d.
HY	< LOQ ^C^	1.36±0.01 ^B^	4.88±0.04 ^A^	< LOQ ^C^	n.d.
IQ	< LOQ ^C^	0.81±0.02 ^B^	2.01±0.10 ^A^	< LOQ ^C^	n.d.
EH	18.78±0.22 ^D^	32.91±0.59 ^B^	104.93±1.10 ^A^	24.81±0.14 ^C^	n.d.
NH	4.52±0.09 ^C^	19.08±0.23 ^B^	43.27±0.55 ^A^	n.d.	n.d.
ER	n.d.	3.98±0.04 ^A^	n.d.	n.d.	n.d.
QU	n.d.	3.80±0.06 ^A^	n.d.	n.d.	n.d.
EC	n.d.	n.d.	4.24±0.03 ^A^	n.d.	n.d.
CI	8.09±0.12 ^C^	41.06±0.85 ^A^	27.79±0.23 ^B^	n.d.	n.d.
*Phenolic fractions*:					
Phenolic acids (TPhA)	171.40±2.37 ^D^	308.47±2.47 ^B^	403.98±3.56 ^A^	248.52±0.44 ^C^	112.52±2.74 ^E^
Flavonoids (TFL)	62.13±0.81 ^D^	103.02±1.78 ^C^	193.93±2.19 ^A^	133.33±0.78 ^B^	1.34±0.02 ^E^
Proanthocyanidins (TPA)	99.94±2.46 ^D^	n.d.	95.26±3.27 ^B^	186.31±8.13 ^A^	27.32±1.07 ^C^
Total polyphenols (TPH)	233.53±3.18 ^D^	411.49±4.24 ^B^	597.91±5.75 ^A^	381.84±1.22 ^C^	113.86±2.76 ^E^
Total polyphenols (TPC)	267.60±2.20 ^D^	598.00±1.30 ^A^	541.30±3.90 ^B^	420.40±3.20 ^C^	135.80±1.20 ^E^

Results are presented as means ± SD (*n* = 3). Different superscript letters (^A–E^) in one row indicate significant differences (*p* < 0.05) in the post-hoc Tukey’s test. LOQ, limit of quantification; n.d. not detected. *Abbreviations of extracts*: ME, methanol-water (7:3, *v/v*) extract; DEF, diethyl ether fraction; EAF, ethyl acetate fraction; BF, *n*-butanol fraction; WR, water residue. *Abbreviations of chemical compounds*: PCA, protocatechuic acid; pHBA, *p*-hydroxybenzoic acid; FAI, ferulic acid isomer; CFA, caffeic acid, NCHA, neochlorogenic acid; CHA, chlorogenic acid; CCHA, cryptochlorogenic acid; cis-CHA, cis-chlorogenic acid; 3,5-diCFQA, 3,5-*O*-dicaffeoylquinic acid; 1,5-diCFQA, 1,5-*O*-dicaffeoylquinic acid; 4,5-diCFQA, 4,5-*O*-dicaffeoylquinic acid; 5-CFSA, 5-*O*-caffeoylshikimic acid; pCA, *p*-coumaric acid; 5-CQA, 5*-O-p*-coumaroylquinic acid; SQ, quercetin 3-*O*-β-sophoroside; RT, rutin; diHQ, quercetin *O*-dihexoside; diHK, kaempferol *O*-dihexoside; HY, hyperoside; IQ, isoquercitrin; EH, eriodictyol *O*-hexosides (two compounds); HN, naringenin *O*-hexoside; ER, eriodictyol; QU, quercetin; EC; (-)-epicatechin; CI, cinchonain I isomers; TPhA, total phenolic acids (determined by HPLC-PDA); TFL, total flavonoids (determined by HPLC-PDA); TPA, total proanthocyanidins (determined by *n*-butanol/HCl assay); TPH, total polyphenols (determined by HPLC-PDA); TPC, total polyphenols (determined by Folin-Ciocalteu assay).

### Effects on carbohydrate-digestive enzymes and non-enzymatic protein glycation

**Fig. 3:**
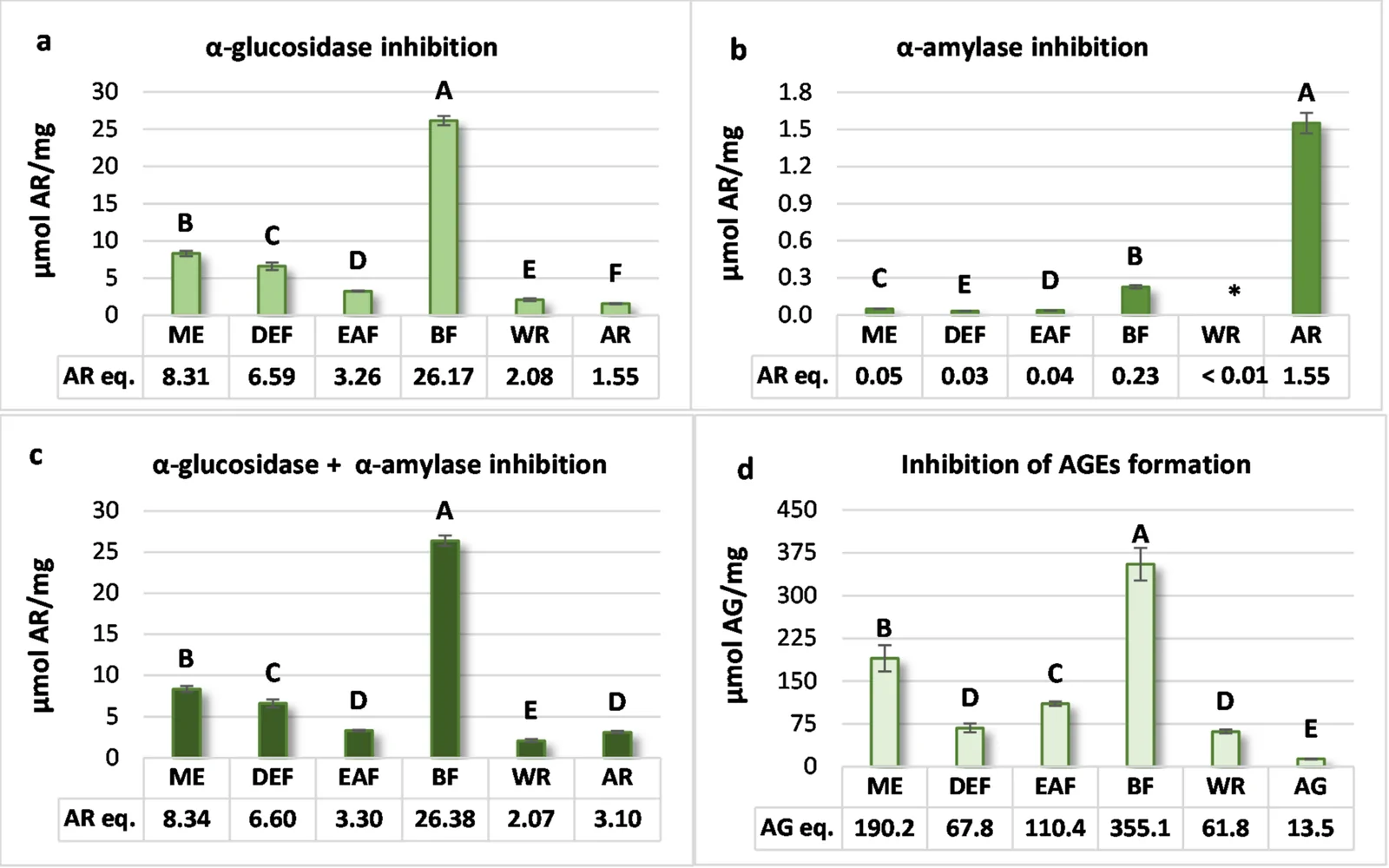
Effects of *S. commixta* leaf extract/fractions on enzymes involved in carbohydrates digestion and influence on non-enzymatic protein glycation: (a) inhibition of α-glucosidase activity calculated as IC_50_ and presented as equivalents of acarbose (AR) activity per extract dry weight (µmol AR/mg); (b) inhibition of α-amylase activity calculated as IC_50_ and presented as equivalents of acarbose (AR) activity per extract dry weight (µmol AR/mg); (c) the sum of α-amylase and α-glucosidase inhibition; (d) inhibition of AGEs (advanced glycation end products) formation calculated as IC_50_ and presented as equivalents of aminoguanidine (AG) activity per extract dry weight (µmol AG/mg). Error bars indicate SD values (*n* = 3). Values on particular charts labelled with the same italics (A-F) did not differ significantly at α = 0.05. ME, methanol-water (7:3, *v/v*) extract; DEF, diethyl ether fraction; EAF, ethyl acetate fraction; BF, *n*-butanol fraction; WR, water residue; * activity too low to be determined in the tested concentration range.

The in vitro analysis of the inhibitory potential of *S. commixta* leaf extracts toward key enzymes involved in carbohydrate digestion showed a stronger inhibitory effect on α-glucosidase compared to the antidiabetic drug acarbose, exhibiting activity approximately 2- to 17-fold higher than the reference standard (Fig. 3a). On the other hand, the inhibition of α-amylase was about 7- to 50-fold weaker than that of acarbose (Fig. 3b). This finding aligns with the tendency described previously that α-glucosidase is significantly more sensitive than α-amylase toward various plant products^34^. Still, the total anti-enzyme activity (sum of α-glucosidase and α-amylase inhibition parameters) of the most active *S. commixta* extracts ME and BF surpassed that of acarbose by about 3 and 9 times, respectively (Fig. 3c).

The study of *S. commixta* leaf extracts influence on non-enzymatic protein glycation revealed about 5- to 26-fold higher inhibitory potential than that observed for aminoguanidine – a prototype therapeutic drug for the prevention of advanced glycation end products (AGEs) formation (Fig. 3d). The most potent activity was revealed for BF, followed by ME.

### Antioxidant effects on human blood plasma components: oxidation and nitration of proteins and lipids

**Fig. 4:**
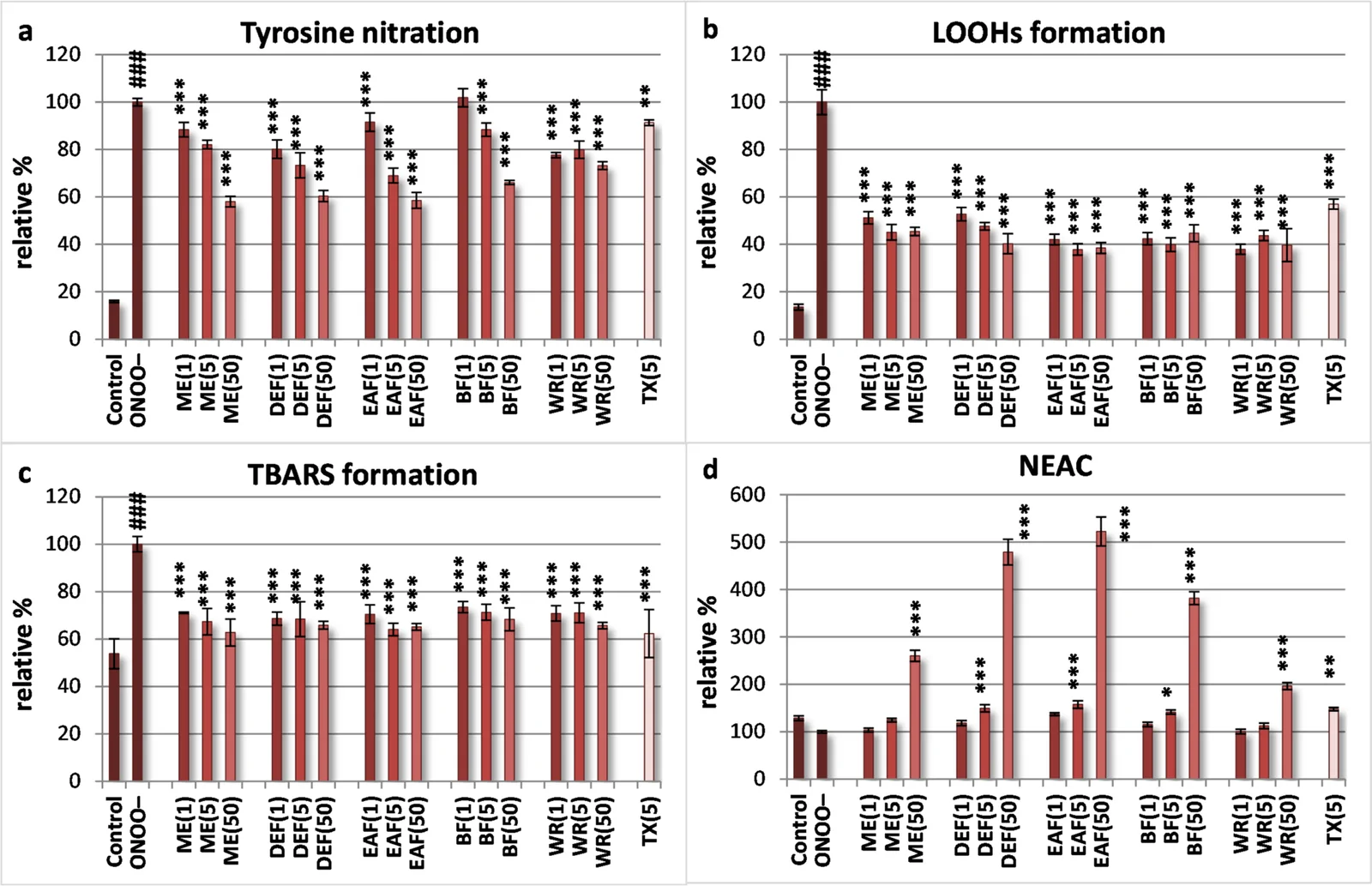
Effects of *S. commixta* leaf extract/fractions on human plasma exposed to oxidative stress: (a) effects on the nitration of tyrosine residues in plasma proteins; (b) effects on the plasma lipids oxidation and the formation of hydroperoxides (LOOHs); (c) effects on the plasma lipids oxidation and the formation of thiobarbituric acid reactive substances (TBARS); (d) effects on the non-enzymatic antioxidant capacity of plasma (NEAC). Results expressed as means ± SE (*n* = 4–8) for repeated measures and presented as relative percentage (assuming the level of the tested parameter in the ONOO^−^-treated plasma without the analytes as 100%). ### *p* < 0.001, for ONOO^−^-treated plasma versus control plasma (without the analytes), and *** *p* < 0.001, ** *p* < 0.01, * *p* < 0.05 for plasma treated with ONOO^−^ in the presence of the tested extract/fraction (1–50 µg/mL) or Trolox (TX, 5 µg/mL) versus ONOO^−^ -treated plasma in the absence of the analytes. ME, methanol-water (7:3, *v/v*) extract; DEF, diethyl ether fraction; EAF, ethyl acetate fraction; BF, *n*-butanol fraction; WR, water residue.

Depending on the extract and its concentration (1–50 µg/mL for extracts, equal to 0.1–30 µg of polyphenols/mL, re-calculated using TPC levels), the ability to inhibit plasma protein nitration (transformation of tyrosine units into 3-nitrotyrosine) reached up to 42%, and the inhibitory effect toward lipid peroxidation was 43–62%, when measured at the step of lipid hydroperoxides (LOOHs), and 26–37%, when measured as thiobarbituric acid-reactive substances (TBARS), the final peroxidation products (Fig. 4a-c). These effects were comparable to or higher than those of Trolox at the corresponding level in terms of extract weight, and even higher when considering the polyphenol content in the extracts. For example, the inhibition of protein nitration by ME at 5 µg of extract/mL (equivalent to 0.65 µg of polyphenols/mL) was about 20%, while it was about 10% for Trolox at 5 µg/mL. The potent antioxidant effects of extracts in plasma were also reflected in their significant ability to enhance the total non-enzymatic antioxidant capacity of plasma (NEAC, FRAP test) (Fig. 4d). In this case, the advantage of fractionation is negligible, and the bioactivity of the crude ME extract is most promising.

### Anti-inflammatory activity: downregulation of pro-inflammatory cytokines in human PBMCs

**Fig. 5:**
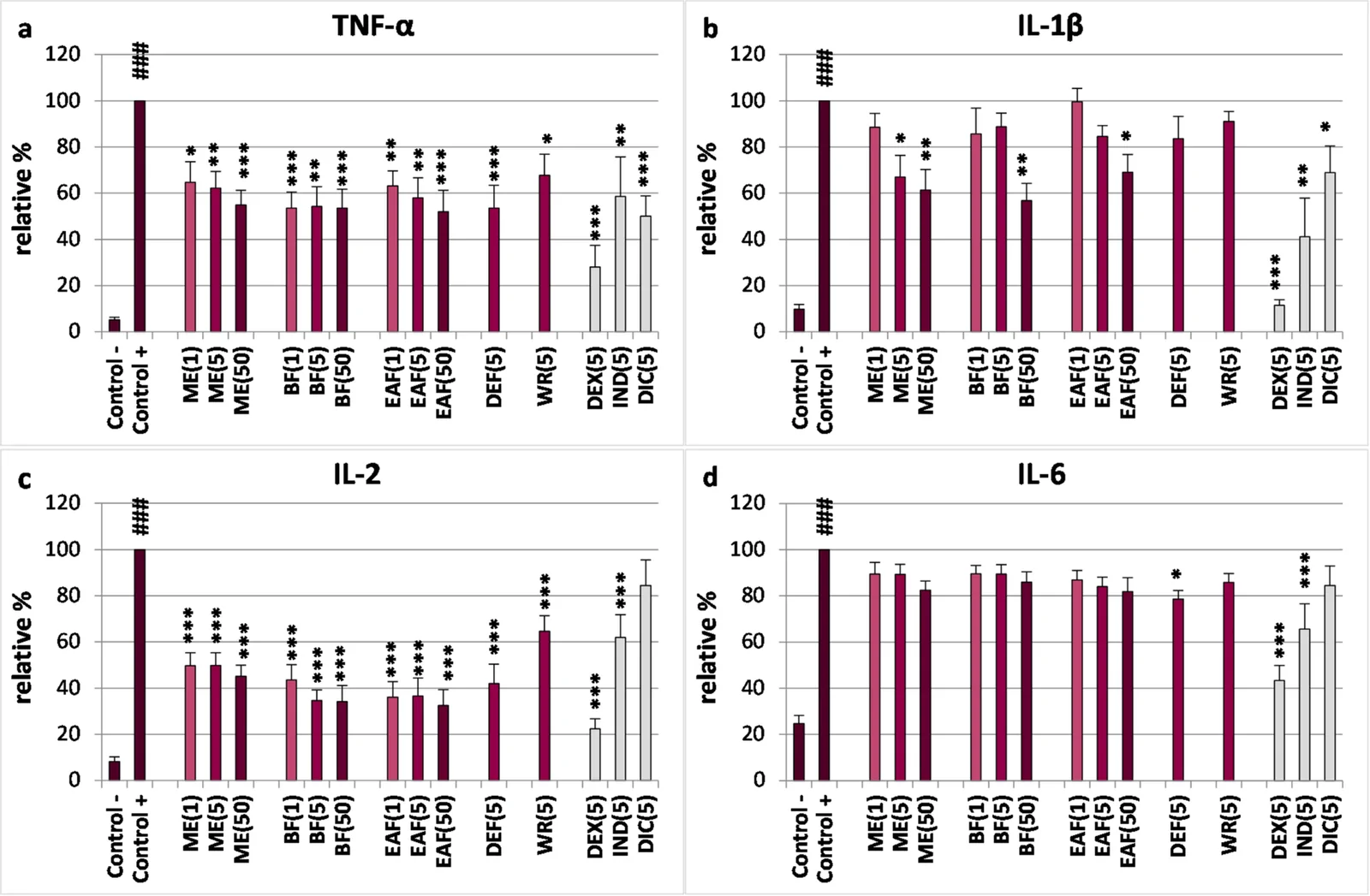
Effects of *S. commixta* leaf extract/fractions on cytokines release from PBMCs: (a) effects on the TNF-α release; (b) effects on the IL-1β release; (c) effects on the IL-2 release; (d) effects on the IL-6 release. Results expressed as means ± SE (*n* = 8–10) assuming the level of the tested parameter in the concanavalin A-treated PBMCs without the analytes as 100%). ### *p* < 0.001, for concanavalin A-treated PBMCs (Control+) versus control PBMCs (Control-, without the analytes), and *** *p* < 0.001, ** *p* < 0.01, * *p* < 0.05 for PBMCs treated with concanavalin A in the presence of the tested extract/fraction (1–50 µg/mL) or positive controls (5 µg/mL) versus concanavalin A-treated PBMCs in the absence of the analytes. ME, methanol-water (7:3, *v/v*) extract; DEF, diethyl ether fraction; EAF, ethyl acetate fraction; BF, *n*-butanol fraction; WR, water residue; DEX, dexamethasone; IND indomethacin; DIC, diclofenac.

The anti-inflammatory potential of *S. commixta* leaf extracts was evaluated based on measuring the release of four pro-inflammatory cytokines, i.e., IL-1β, IL-2, IL-6, and TNF-α, from PBMCs stimulated with concanavalin A (Fig. 5). The extracts were found to be potent inhibitors of IL-2 and TNF-α secretion, with a strong and statistically significant downregulation observed across the entire concentration range (1–50 µg/mL, *p* < 0.05). The activity toward these cytokines was similar for all extracts, with a slight advantage for BF and EAF fractions in the case of IL-2. The release of IL-1β was also reduced, but with varying effectiveness depending on the extract: the effect of ME was statistically significant (*p* < 0.05) at 5–50 µg/mL, while the BF and EAF fractions were active only at 50 µg/mL. On the contrary, all the extracts were found inactive toward IL-6, except for DEF. Compared to reference drugs, the activity of *S. commixta* leaf extracts was similar or higher than that observed for indomethacin and diclofenac (nonsteroidal anti-inflammatory drugs), but lower than that of dexamethasone (a corticosteroid); however, dexamethasone resolves inflammation through a different mechanism than polyphenols^35^.

### Influence on PBMCs viability

Cellular biocompatibility of the extracts within the tested concentration range was evidenced by the lack of significant differences (*p* > 0.05) between the viability of PBMCs treated with *S. commixta* leaf extracts (1–50 µg/mL) and untreated cells (Supplementary Fig. S1 online).

### Phenolic contribution to the biological effects: correlation studies

**Fig. 6:**
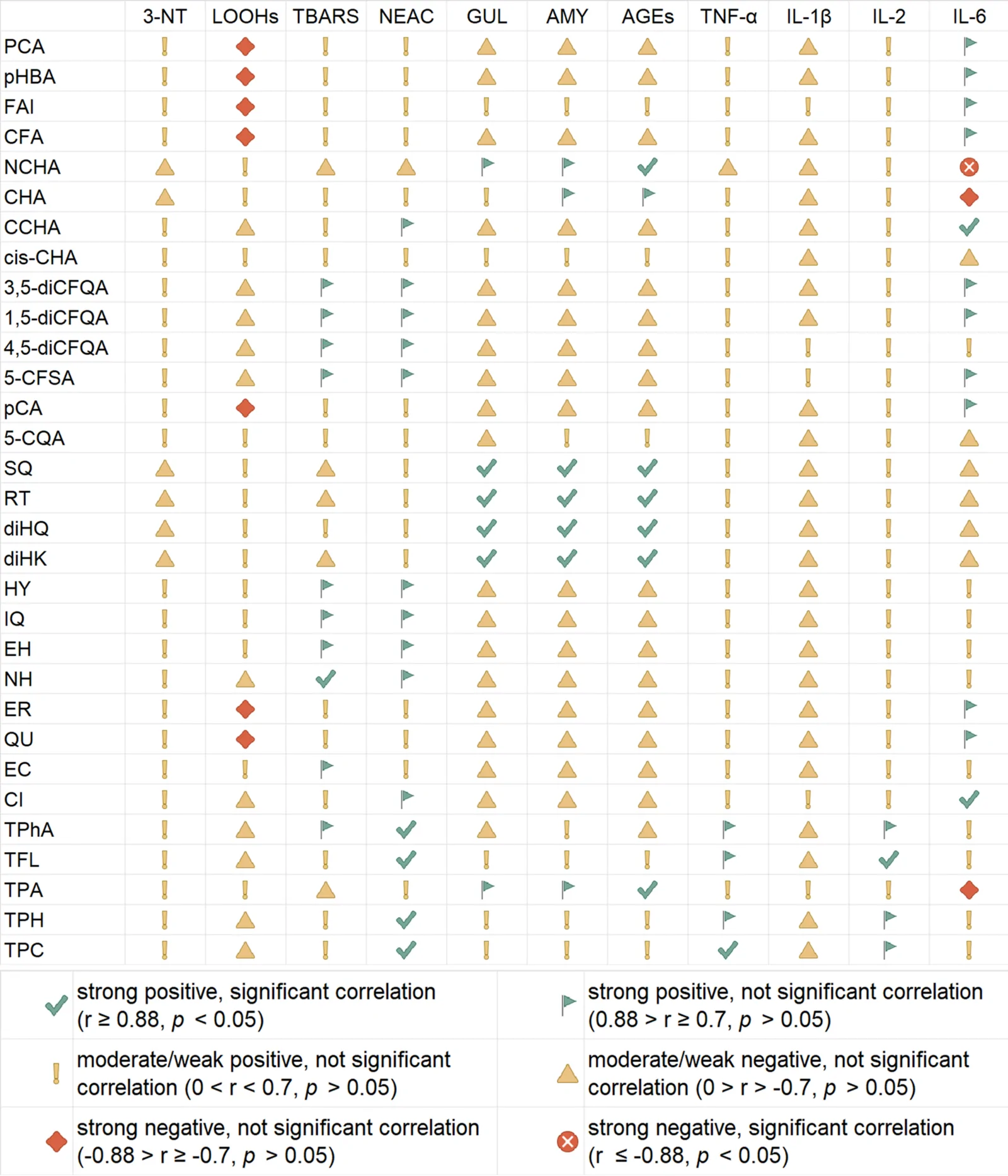
The correlation diagram between phenolic variables and activity parameters. Activity and concentration parameters according to Table 1; Figs. 3, 4 and 5. For statistical analysis, the activity in human plasma model and cellular studies were calculated as percentage of decrease (for 3-NT, LOOHs, TBARS, IL-1β, IL-2, IL-6, and TNF-α) or increase (for NEAC) of the tested parameter in the plasma treated with ONOO^−^ in the presence of the investigated extracts versus ONOO^−^ -treated plasma in the absence of the analytes. *Abbreviations of chemical compounds*: PCA, protocatechuic acid; pHBA, *p*-hydroxybenzoic acid; FAI, ferulic acid isomer; CFA, caffeic acid, NCHA, neochlorogenic acid; CHA, chlorogenic acid; CCHA, cryptochlorogenic acid; cis-CHA, cis-chlorogenic acid; 3,5-diCFQA, 3,5-*O*-dicaffeoylquinic acid; 1,5-diCFQA, 1,5-*O*-dicaffeoylquinic acid; 4,5-diCFQA, 4,5-*O*-dicaffeoylquinic acid; 5-CFSA, 5-*O*-caffeoylshikimic acid; pCA, *p*-coumaric acid; 5-CQA, 5*-O-p*-coumaroylquinic acid; SQ, quercetin 3-*O*-β-sophoroside; RT, rutin; diHQ, quercetin *O*-dihexoside; diHK, kaempferol *O*-dihexoside; HY, hyperoside; IQ, isoquercitrin; EH, eriodictyol *O*-hexosides (two compounds); NH, naringenin *O*-hexoside; ER, eriodictyol; QU, quercetin; EC; (-)-epicatechin; CI, cinchonain I isomers; TPhA, total phenolic acids (determined by HPLC-PDA); TFL, total flavonoids (determined by HPLC-PDA); TPA, total proanthocyanidins (determined by *n*-butanol/HCl assay); TPH, total polyphenols (determined by HPLC-PDA); TPC, total polyphenols (determined by Folin-Ciocalteu assay). *Abbreviations of activity parameters*: 3-NT, effects on the nitration of tyrosine residues; LOOHs, effects on the plasma lipids oxidation and the formation of hydroperoxides; TBARS, effects on the plasma lipids oxidation and the formation of thiobarbituric acid reactive substances; NEAC, effects on the non-enzymatic antioxidant capacity of plasma; GLU, α-glucosidase inhibition; AMY, α-amylase inhibition; AGEs, inhibition of the advanced glycation end products formation; IL-1β, IL-2, IL-6, TNF-α, inhibition of cytokines release.

To evaluate the relationship between phenolic composition and activity parameters, a correlation study was performed. It was carried out based on the quantitative results obtained for individual phenolics (compounds that accounted for 82–97% of TPH), phenolic fractions (TPhA, TFL, TPA, TPH, TPC levels), as well as 11 activity parameters (Fig. 6). For detailed correlation parameters, see Supplementary Table S2 online.

As a result, a strong, positive correlation was found between TPA, NCHA, SQ, RT, diHQ, diHK, and antidiabetic activity (inhibition of α-glucosidase, α-amylase, and AGEs). On the other hand, the antioxidant parameters (TBARS and NEAC) positively correlated with individual phenolic acids (CCHA, 5-CFSA, 3,5-diCFQA, 1,5-diCFQA, 4,5-diCFQA, 5-CQA, cis-CHA), flavanones (EH, NH), flavonols (IQ, HY), flavanols (EC), and flavalignans (CI1, CI2); however, they were mostly not significant (*p* > 0.05), with statistical significance observed only for NEAC vs. total phenolic acids (TPhA), total flavonoids (TFL), and total phenolics (TPH and TPC), as well as TBARS and NH (*p* < 0.05). Also, a strong, negative but not significant correlation was observed for LOOHs and simple phenolic acids (PCA, pHBA, FAI, CFA, pCA) and flavonoid aglycones (ER, QU). As for anti-inflammatory parameters, the downregulation of IL-2 and TNF-α positively correlated with the total levels of flavonoids and phenolic acids; while IL-6 corresponded mainly to the content of different phenolic acids and flavonoid aglycones. In the case of IL-1β and 3-NT, no correlation was observed with any of the phenolic components; therefore, the proportion between different polyphenols or their synergy may be vital for these activities (Fig. 6).

## Discussion

In this study, we aimed to evaluate the potential of *S. commixta* leaves as a source of biologically active compounds, with particular emphasis on phenylpropanoids exhibiting antioxidant, anti-inflammatory, and antidiabetic properties. To achieve this, a detailed characterisation of the phytochemical composition was undertaken, as it is essential for identifying the major metabolite groups potentially responsible for the biological properties of the plant and for distinguishing *S. commixta* from other species within the *Sorbus* genus.

For this purpose, we applied fractionated extraction, a method that both concentrates bioactive products and separates complex plant matrices into groups of molecules. This separation is beneficial for the identification of a broader range of compounds, enables more precise evaluation of activity-related components, and aids in the identification of markers relevant for the quality control^4,16^.

Indeed, by using fractionated extraction, we were able to identify 78 phenolic compounds (Fig. 2, Supplementary Table S1), in contrast to only 13 phenolics that had been reported previously in *S. commixta* leaves^4,5,8^, revealing a significantly more complex composition of this plant material. Moreover, 30 compounds were detected for the first time in the genus *Sorbus*. They were proanthocyanidin hexosides (8 new compounds), caffeic acid derivatives (16 new constituents, mainly hexosides), flavanones (3 compounds), flavalignans (2 compounds, derivatives of cinchonain I isomers), and one quercetin triglycoside (Fig. 2, Supplementary Table S1).

The glycosylation of polyphenols (phenolic acids and flavonoids) yields derivatives with improved stability and enhanced biological properties, especially in the case of relatively unstable flavanols^36,37^. For instance, the intestinal absorption (rat model) of (+)-catechin 3ʹ-*O*-β-D-glucopyranoside rose from 16% to 28% compared to (+)-catechin, with about 5-fold higher thermal and pH stability observed for the glucoside^37^. In this light, *S. commixta* leaf extracts, containing a diversity of glycosylated polyphenols, may be an advantageous source of bioactive phenolics.

Importantly, *S. commixta* stands out from over thirty *Sorbus* species studied so far^8^ due to its rich and diversified fraction of flavanones. While two eriodictyol hexosides (peaks no. 47, 48), naringenin hexoside (peak no. 62), and eriodictyol aglycone (peak no. 77) were among the dominant components of the studied *S. commixta* leaf extracts (Fig. 2, Supplementary Table S1), only traces of one flavanone (eriodictyol hexoside or eriodictyol glucuronide) were previously found in the fruits and flowers of *S. aucuparia*, fruits of *S. torminalis*, and fruits of *S. discolor*^3,26^.

Considering the known beneficial health effects of some flavanones, their presence in *S. commixta* leaves might be promising. For instance, clinical trials of hesperidin have revealed its ability to reduce oxidative damage to lipids and DNA in patients with diabetes, as well as to improve endothelial function and to reduce circulating biomarkers of inflammation in subjects with metabolic syndrome^38^. Furthermore, due to their rare occurrence in *Sorbus*, the target compounds (primarily peaks no. 47, 48, 62) may be considered taxonomic markers of the plant, useful in authentication tests during quality control.

The quantitative HPLC-PDA analysis also confirmed the possibility of high biological potential of the tested extracts (Table 1). The observed phenolic abundancy (TPH from 233.5 mg/g dw for ME to 380.18–597.9 mg/g dw for BF, DEF, and EAF) places the *S. commixta* leaves at the top of previously tested leaves, flowers, fruits, and barks of over thirty *Sorbus* species^6–8,13^. Moreover, considering that the TPH levels in the commercial extracts of olive leaves and grape seeds, known as excellent sources of polyphenols, are usually about 200–400 mg/g dw and 800–900 mg/g dw^39,40^, our results also demonstrated the relevant value of *S. commixta* leaves in general.

This can also be valid when comparing the contents of different phenolic groups in *S. commixta* leaves/extracts and the corresponding contents in other phenolic-abundant plants with well-established use in medicine. For instance, the caffeoylquinic acids content was up to 276.2 mg/g dw in EAF vs. average TCHA is 268 mg/g dw in commercial green coffee bean extracts; flavanones content was up to 148.7 mg/g dw in EAF vs. approximately 66.5–86.5 mg/g dw in the willow bark or liquorice root dry extracts; while total proanthocyanidins content calculated per dry leaf was about 30 mg/g dw vs. about 35 mg/g in the grape seeds^39–44^.

The distinctive phytochemical profile of *S. commixta* leaves, characterised by high levels of polymeric proanthocyanidins, caffeoylquinic acids, flavonols, and rare flavanones, indicates their considerable potential for multidirectional biological activity. However, compositional data alone are insufficient to establish their functional relevance. Therefore, their effects were examined in vitro/ex vivo in relation to key biochemical and cellular processes associated with oxidative and nitrative stress, inflammation, carbohydrate metabolism, and protein glycation. Investigating the potential of *S. commixta* leaf extracts to modulate some molecular mechanisms involved in these processes provided further insight into composition-activity relationships and supported the identification of compounds associated with the observed effects (as putative active markers).

The selected biological assays addressed interconnected pathways central to oxidative stress-driven metabolic dysfunction while also reflecting the predominant activities previously attributed to *Sorbus* plants and *Sorbus*-typical polyphenolic constituents. Lipid peroxidation and protein nitration represent ROS/RNS-mediated molecular damage that perturbs redox homeostasis and promotes downstream signalling, including activation of pro-inflammatory cascades and cytokine release. In parallel, non-enzymatic protein glycation and subsequent formation of advanced glycation end-products (AGEs) are facilitated under oxidative conditions and further amplify inflammatory and redox disturbances. Postprandial hyperglycaemia, modulated by α-amylase and α-glucosidase activity, acts as an upstream metabolic trigger that aggravates both glycoxidative stress and inflammatory responses. This integrated selection of endpoints therefore enables the assessment of complementary biochemical and cellular processes that jointly contribute to oxidative, inflammatory, and metabolic dysregulation implicated in the pathogenesis of atherosclerosis, diabetes, or rheumatism, i.e., conditions traditionally treated with plant polyphenols, including those of *Sorbus* origin^8,24,45,46^.

To test the potential protective effects of the tested leaf extracts against oxidative/nitrative stress-derived damage to biomolecules, a human plasma ex vivo model was applied. The stress conditions were induced by ONOO^–^, one of the most aggressive ROS/RNS generated in humans. Notably, despite differences in composition and phenolic concentration, all tested extracts exhibited similar, significant protective effects against oxidative/nitrative damage to plasma proteins and lipids (inhibition up to 42% and 62%, respectively) (Fig. 4). In addition, the significant ability to enhance the NEAC of plasma (Fig. 4) was observed for all samples. All these results may partially legitimise traditional recommendations for *S. commixta* in managing atherosclerosis, gastrointestinal disorders, etc^8^., as lipid and protein oxidation are key hallmarks of these disorders^47^. The mechanisms that may be behind the observed activity in the plasma model may include scavenging multiple oxidants (ONOO^‒^ itself and ONOO^‒^-derived secondary radicals, i.e., ^•^OH, NO^•^, O_2_^•–^, ^•^NO_2_, CO_3_^•–^, etc.) that were previously reported for various herbal materials from *Sorbus* species, including *S. commixta* bark and stems^8,48–50^. Moreover, alleviation of metal-induced oxidation of biomolecules or direct protection of plasma molecules by creating reversible or irreversible chemical bonds may also be involved^8,48–50^.

AGEs are formed in the non-enzymatic reaction between carbonyl groups of reducing sugars and amino groups of proteins, or as by-products of glucose autoxidation or lipid peroxidation^46^. Early glycation products, including glyoxal, methylglyoxal, and Amadori products, are transformed into more stable AGEs, which may cross-link with proteins or DNA and subsequently alter the organ structure and function. During the progression of diabetes, AGEs may directly impair pancreatic β-cells or modify insulin, contributing to insulin resistance. However, they may also accumulate in vascular, heart, liver, or kidney tissues, leading to diabetic complications, e.g., non-alcoholic fatty liver disease, vascular diseases, intestinal enteropathy, etc^46^.

There are a few known inhibitors of AGEs, but their use is limited due to harmful side effects (e.g., discontinued clinical trials of aminoguanidine) or an insufficient number of studies confirming their effectiveness (e.g., pyridoxamine and its derivatives)^51^. There were also reports indicating that some drugs used in other diseases, such as angiotensin inhibitors, may reduce AGEs, albeit at a modest level only^46^. Therefore, in the quest for effective AGEs inhibitors, increased attention has been given to plant-derived substances.

The anti-glycation potential was previously noted for, e.g., *S. commixta* 80% methanol fruit extract; however, its activity was around 15-fold lower than that observed for aminoguanidine^52^. Hence, the anti-glycation potential observed in this study for *S. commixta* leaf extracts (5-26-fold higher than that of aminoguanidine, Fig. 3) seems to be remarkable. Indeed, based on the literature, *S. commixta* leaves may represent a potentially promising plant material compared with many other plant extracts. For instance, i.e., *Trigonella foenum-graecum* seed extract or *Gymnema sylvestre* leaf extract, known antidiabetic herbal agents, have the anti-glycation capacity comparable to or at best a few times higher than aminoguanidine^53,54^.

While direct inhibition of AGEs formation may entail benefits for various systems and tissues, an equally significant importance can be attributed to lowering the sugar level, i.e., the removal of the causative factor of glycation stress. Glycoside hydrolases, such as α-amylase and α-glucosidase, are key enzymes responsible for carbohydrate digestion in humans. The α-amylase catalyses the hydrolysis of α-1→4 glycosidic bonds in polysaccharides to oligosaccharides and disaccharides that undergo further digestion to monosaccharides by α-glucosidases – the specific enzymes situated in the brush border of the small intestine^19^. Most commercially available glucosidase inhibitors, such as acarbose, are able to effectively decrease postprandial glucose levels with no hypoglycaemia risk, but they are also responsible for some adverse gastrointestinal side effects^55^. Therefore, the use of α-amylase or α-glucosidase inhibitors of plant origin, which may be devoid of the adverse effects associated with synthetic drugs, may be a step forward in safer diabetes management.

Previously, the anti-enzyme activity was tested for the 70% acetone fruit extracts of *S. commixta* and *S. commixta var. rufoferruginea*, and the observed effectiveness towards α-glucosidase and α-amylase, respectively, was about 2- and 20-fold higher, and 14- and 38-thousand times lower than reported for acarbose^56^. In this context, while the potential of *S. commixta* leaf extracts towards α-glucosidase seems to be at a similar level (2-17-fold stronger inhibition compared to the acarbose), their efficiency towards α-amylase, although weak (7-50-fold weaker than that of acarbose), is still many times stronger than that of related plant materials (Fig. 3). The inhibitory potential of the tested samples toward glucosidases is also noticeable compared to other plant species, including those with documented anti-diabetic applications. For example, the α-glucosidase inhibitory activity of *Morus alba* leaf extract was about 4.5 times higher than that of acarbose^57^.

Inflammation is a complex response involving different immune cells that engage various signalling pathways, associated with the development and progression of many pathological conditions, including diabetes-related complications affecting the cardiovascular system, gastrointestinal tract, kidney, and liver^45^. Given the central role of cytokines in pro-inflammatory signalling, targeted therapies against specific mediators (e.g., TNF-α or IL-1β) have been widely investigated^58^. However, the complexity of inflammatory pathways suggests that modulation of multiple targets may be advantageous. In this context, plant-derived extracts, composed of diverse bioactive constituents, may contribute to a broader regulation of cytokine-mediated responses.

The significant (*p* < 0.05 − 0.001) inhibition of TNF-α, IL-1β, and IL-6 expression was previously observed in cartilage tissue of rats with osteoarthritis treated with 100–300 mg/kg of 30% ethanol stem extract of *S. commixta*, in the rat serum, and in lipopolysaccharide (LPS)-activated RAW264.7 rat macrophages treated with 30–300 µg/mL of the same extract^10^. As in our study (Fig. 5), despite the different plant materials (leaves vs. stems), the intensity of inhibition was similar, i.e., superior activity toward TNF-α, followed by IL-1β, and the weakest impact on IL-6. In contrast, the high impact on the IL-6 level was observed for 80% ethanol extract from *S. commixta* twigs (in primary normal human dermal fibroblasts and human adult low calcium high temperature keratinocyte cells, HaCaT, at 12.5–100 µg/mL) and water extract from *S. commixta* fruits (in BV-2 microglia cells, at 25–100 µg/mL)^11,59^. Moreover, there is also one study reporting no effect of 70% ethanol extract of *S. commixta* (analysed plant part is not known) at 100–300 µg/mL on TNF-α release from LPS-stimulated RAW264.7 cells^60^. On top of that, all of the *S. commixta* extracts tested previously (from stems, twigs, and fruits) also affected different inflammatory-related signalling pathways, such as MAPK, NF-κB, and cyclooxygenase 2^11,59^, suggesting various anti-inflammatory mechanisms involved and opening the door to further research on the *S. commixta* leaves.

In summary, the accumulated previous research suggests that *S. commixta* is a source of potent anti-inflammatory agents; however, their anti-inflammatory effectiveness depends on the plant part and the type of extract (and therefore its composition), as well as the study model applied. In this context, the present research documents the promising potential of *S. commixta* leaves and leaf extracts, which are able to significantly modify the pro-inflammatory functions of human PBMCs ex vivo at relatively low concentration levels of 1–5 µg/mL, and with a strength similar or higher than that observed for nonsteroidal anti-inflammatory drugs (indomethacin and diclofenac).

Finally, keeping in mind the susceptibility of plant materials to compositional variability and the influence of phenolic profiles on the biological effects of natural products, the next step of the present research was to evaluate which of the identified constituents were responsible for the observed activity of *S. commixta* leaf extracts. Correlation analysis using Pearson’s coefficient indicated associations of polymeric proanthocyanidins, flavonol diglycosides, caffeoylquinic acids, and monoglycosides of flavonols and flavanones with the tested biological effects (for details, see results section, Fig. 6, and Table S2).

Indeed, compounds from all the mentioned phenolic groups are known for their antidiabetic, anti-inflammatory, and antioxidant properties, confirmed in pre-clinical or clinical studies. The inhibition of cytokine secretion, scavenging of free radicals, the ability to increase the levels of endogenous antioxidant enzymes, to reduce the oxidation or glycation of functional biomolecules, and to lower blood glucose and pro-oxidant markers in the tissues are among the main effects reported for different proanthocyanidins, flavonols, flavanones, and caffeoylquinic acids^38,50,61–64^. For example: (1) proanthocyanidins from grape seeds were reported to lower blood glucose, decrease the level of oxidative stress markers (ROS, LOOHs, malondialdehyde) and inflammatory cytokines (TNF-α, IL-1 β, INF-α), as well as increase the level of antioxidant enzymes (catalase, glutathione peroxidase, glutathione reductase, superoxide dismutase) in the tissues of rats with induced pancreatitis^61^; (2) eriodyctiol treatment decreased the level of TNF-α, adhesion molecules, and endothelial NO synthase in the retina, and TBARS in the plasma of diabetic rats^62^; (3) isoquercitrin supplementation decreased the IL-1β level and the ‘z score’ for inflammation (calculated based on seven parameters: the levels of TNF-α, IL-1β, IL-6, IL-8, C-reactive protein, serum amyloid A, and soluble intercellular adhesion molecule-1) in the prehypertensive adults^63^. Moreover, the biological effectiveness of phenolic fractions is often enhanced by the synergy of different compounds, and indeed this effect was noticed between different phenolic acids and flavonoids in terms of antioxidant, anti-TNF-α, anti-IL-1β, and anti-IL-6 activity^50,65,66^.

The multidirectional activity of different phenolics and the synergistic effects between them contribute to the potential therapeutic role of plant products in addressing cardiovascular diseases, diabetes, metabolic syndrome, and a broad range of diseases related to oxidative stress and inflammation. In this context, a complex fraction of polyphenols present in the *S. commixta* leaves added a significant beneficial synergy factor to their antioxidant, antidiabetic, and anti-inflammatory potential.

However, while the current study provides some mechanistic evidence supporting the biological potential of *S. commixta* leaves under in vitro and ex vivo conditions, the conclusions should be interpreted with caution. The experimental design does not allow for direct extrapolation of the results to in vivo efficacy or therapeutic outcomes, as it does not consider factors like absorption, metabolism, bioavailability, or tissue distribution. On the other hand, the concentration range applied in vitro (1–50 µg/mL) was selected based on literature data indicating that dietary phenolics typically reach low micromolar plasma levels after oral intake. For instance, flavonol glucosides yield peak quercetin concentrations of 2.1–7.6 µM (0.6–2.3 µg/mL), while caffeoylquinic acids may reach up to 14.8 µM (5.2 µg/mL)^21–23^. Consequently, the lower concentrations applied in this study (1–5 µg/mL) may reflect physiologically achievable systemic exposure. Additionally, literature reports indicate that 90–95% of ingested phenolics reach the large intestine, where they can accumulate to millimolar concentrations^67^. Therefore, the effects observed in vitro at concentrations ≥ 50 µg/mL may be relevant to the gastrointestinal environment. Nevertheless, these considerations stem from previously published studies rather than being directly demonstrated within the current experiments. Therefore, further in vivo investigations are essential to validate pharmacokinetics and physiological relevance, as well as to confirm whether the activities observed in vitro correspond to in vivo effects. Overall, the findings provide a solid basis supporting the biological potential of *S. commixta* polyphenols and justify continued investigation in more advanced experimental models.

## Conclusions

This study provides a comprehensive phytochemical and bioactivity-based evaluation of *S. commixta* leaves as a previously underexplored, high-value source of polyphenolic compounds. A total of 78 constituents were identified, encompassing a broad spectrum of polyphenols, including rare flavanones and flavalignans, which were associated with the observed multifunctional bioactivity profile. The crude water-alcoholic leaf extract exhibited a high extraction yield (30%) and substantial polyphenolic content (268 mg GAE/g dw), confirming the suitability of this plant material for efficient recovery of bioactive metabolites. Subsequent fractionation further enhanced the content of active phytochemicals up to 598 mg GAE/g dw. This value reaches polyphenol content reported for, e.g., olive leaf, green coffee bean, and grape seed extracts (known industrial sources of polyphenols), as well as previously investigated *Sorbus* materials, including *S. commixta* bark. Functionally, fractionation markedly improved bioactivity, most notably in the *n*-butanol fraction, which showed a 3-fold increase in inhibition of α-glucosidase and AGEs formation compared to the crude extract and revealed substantially higher antidiabetic potential in comparison to reference drugs (acarbose and aminoguanidine; up to 17- and 26-fold, respectively). In addition, both crude extract and enriched fractions exerted strong protective effects against ROS/RNS-induced damage to proteins and lipids in human plasma ex vivo and significantly attenuated pro-inflammatory cytokine release from PBMCs, without detectable cytotoxicity.

Overall, these findings indicate that *S. commixta* leaves may represent a valuable source of multifunctional polyphenol-rich preparations within the *Sorbus* genus, showing favourable chemical composition and biological activity compared with several traditionally utilised plant parts. These results suggest the possibility of using the leaves in the development of standardised bioactive preparations with potential relevance to oxidative stress, inflammation, and glycation-associated processes involved in metabolic disorders. However, future studies should prioritise in vivo validation and further research of the mechanisms of action, particularly in relation to key molecular targets within redox and glucose-regulatory pathways.

## Supplementary Information

Below is the link to the electronic supplementary material.


Supplementary Material 1


## Data Availability

Data will be made available on request.
