# Supplementary material for: Polyphenol profiling and multi-target antioxidant, anti-inflammatory and antidiabetic activity of Sorbus commixta Hedl. leaves
Source: Sci Rep. 2026 Jun 13;16:27347. doi: 10.1038/s41598-026-57556-5 (PMC13530270; doi:10.1038/s41598-026-57556-5)
Supplement: Supplementary file 1 [file 41598_2026_57556_MOESM1_ESM.docx]

**Supplementary materials**

**Polyphenol profiling and multi-target antioxidant, anti-inflammatory and antidiabetic activity of *Sorbus commixta* Hedl. leaves**

**Magdalena Rutkowska^1*^, Joanna Kolodziejczyk-Czepas^2^, Oleksandra Liudvytska^2^, Piotr Michel^1^, Monika A. Olszewska^1^**

^1^ Department of Pharmacognosy, Faculty of Pharmacy, Medical University of Lodz, 1 Muszynskiego St., 90-151 Lodz, Poland

^2^ Department of General Biochemistry, Faculty of Biology and Environmental Protection, University of Lodz, Pomorska 141/143, 90-236 Lodz, Poland.

*Correspondence: [magdalena.rutkowska@umed.lodz.pl](mailto:magdalena.rutkowska@umed.lodz.pl)

**Supplementary Table S1.** Qualitative composition of *S. commixta* leaf extracts/fractions.

| No. | Analyte | *R_t_* (min) | UV λ_max_ (nm) | [M–H]^–^ (m/z) | MS^2^/MS^3^ fragment ions | Extract | References |
| --- | --- | --- | --- | --- | --- | --- | --- |
| 1 | Protocatechuic acid hexoside | 4.5 | 255 | 315 | 153(100) | ME, WR | [1] |
| 2 | Caffeic acid hexoside | 5.0 | 215, 325 | 341 | 179(100) | EAF, BF | [1] |
| 3 | Protocatechuic acid (PCA) *^ab^* | 5.3 | 259, 294 | 153 |  | DEF |  |
| 4 | 3-*O*-Caffeoylquinic acid (NCHA, neochlorogenic acid) *^ab^* | 5.4 | 216, 324 | 353 | 191(100), 179(42), 135(5) | ME, DEF, EAF, BF, WR | [2] |
| 5 | 5-*O*-Caffeoylquinic acid hexoside | 6.0 | 216, 325 | 515 | 353(100), 191(28) | ME, WR | [1] |
| 6 | (Epi)catechin-B-(epi)catechin | 6.7 | 279 | 577 | 451(26), 425(100), 407(35) | ME, EAF, BF | [1] |
| 7 | (Epi)catechin-B-(epi)catechin | 8.0 | 279 | 577 | 425(100), 407(50), 289(18) | EAF | [1] |
| 8 | *p*-Hydroxybenzoic acid (pHBA) *^ab^* | 8.4 | 255 | 137 |  | DEF |  |
| 9 | (Epi)catechin-B-(epi)catechin | 8.4 | 279 | 577 | 425(100), 407(55), 289(11) | EAF, BF | [1] |
| 10 | 5-*O*-Caffeoylquinic acid (chlorogenic acid, CHA) *^ab^* | 9.1 | 216, 325 | 353 | 191(100), 179(5) | ME, DEF, EAF, BF, WR | [2] |
| 11 | (+)-Catechin *^a^* | 9.7 | 278 | 289 | 245(100), 205(21) | EAF |  |
| 12 | 4-*O*-Caffeoylquinic acid (CCHA, cryptochlorogenic acid) *^ab^* | 10.6 | 216, 325 | 353 | 191(50), 179(49), 173(100) | ME, DEF, EAF, BF, WR | [2] |
| 13 | Caffeic acid acetyl-hexoside *^c^* | 12.2 | 215, 325 | 383 | 341(13), 323(21), 179(100) | ME, DEF, EAF, BF | [3] |
| 14 | Caffeic acid (CFA) *^ab^* | 12.6 | 215, 323 | 179 |  | DEF |  |
| 15 | (Epi)catechin-B-(epi)catechin | 12.9 | 279 | 577 | 425(100), 407(54), 289(30) | EAF | [1] |
| 16 | Procyanidin B2 *^a^* | 13.5 | 279 | 577 | 451(18), 425(100), 407(33) | EAF |  |
| 17 | Caffeoylmalic acid *^c^* | 14.1 | 215, 325 | 295 | 179(100), 135(43) | ME, EAF | [4] |
| 18 | cis-5-*O*-Caffeoylquinic acid (cis-CHA, cis-chlorogenic acid) *^c^* | 14.5 | 216, 325 | 353 | 191(100), 179(4) | ME, EAF, BF, WR | [5] |
| 19 | Caffeic acid acetyl-hexoside *^c^* | 14.7 | 215, 325 | 383 | 341(8), 323(23), 179(100) | ME, DEF, EAF, BF | [3] |
| 20 | 5*-O-p*-Coumaroylquinic acid (5-CQA) | 15.5 | 207, 311 | 337 | 191(100), 163(8) | ME, EAF, BF | [2] |
| 21 | (–)-Epicatechin (EC) *^a^* | 16.0 | 279 | 289 | 245(100), 205(20) | EAF |  |
| 22 | Caffeic acid acetyl-hexoside *^c^* | 17.1 | 215, 325 | 383 | 341(5), 323(20), 179(100) | ME, DEF, EAF, BF | [3] |
| 23 | 5-*O*-Caffeoylshikimic acid (5-CFSA) | 17.6 | 211, 325 | 335 | 291(20), 179(100), 135(18) | ME, DEF, EAF | [1] |
| 24 | (Epi)catechin-B-(epi)catechin hexoside *^c^* | 18.1 | 279 | 739 | 587(100), 407(88), 289(61) | EAF | [6] |
| 25 | Caffeic acid acetyl-hexoside *^c^* | 18.8 | 215, 325 | 383 | 341(3), 323(9), 179(100) | ME, DEF, EAF, BF | [3] |
| 26 | Caffeic acid hexoside derivative *^c^* | 19.6 | 215, 325 | 425 | 341(100), 323(84), 179(41) | DEF, EAF | [1] |
| 27 | Quercetin *O*-rhamnosyl-hexosyl-hexoside *^c^* | 19.7 | 255, 353 | 771 | 609(100)*^d^*, 301(100)*^e^* | BF | [1] |
| 28 | Vanillic acid *^a^* | 20.0 | 260, 293 | 167 |  | DEF |  |
| 29 | (Epi)catechin-B-(epi)catechin-B-(epi)catechin | 20.1 | 279 | 865 | 713(98), 695(100), 577(83) | EAF | [1] |
| 30 | *p*-Coumaric acid (pCA) *^ab^* | 20.9 | 293 | 163 |  | DEF |  |
| 31 | Quercetin *O*-dihexoside | 21.0 | 255, 353 | 625 | 463(10), 445(30), 301(100) | ME, BF | [1] |
| 32 | (Epi)catechin-B-(epi)catechin hexoside *^c^* | 21.2 | 279 | 739 | 587(100), 407(77), 289(28) | EAF | [6] |
| 33 | (Epi)catechin-B-(epi)catechin-B-(epi)catechin | 21.2 | 279 | 865 | 577(78), 695(100), 407(49) | EAF | [1] |
| 34 | Quercetin 3-*O*-β-sophoroside (SQ) *^ab^* | 21.9 | 255, 353 | 625 | 463(13), 445(39), 301(100) | ME, EAF, BF, WR |  |
| 35 | Quercetin *O*-dihexoside (diHQ) | 22.4 | 255, 352 | 625 | 463(24), 445(40), 301(100) | ME, EAF, BF | [1] |
| 36 | (Epi)catechin-B-(epi)catechin | 22.5 | 279 | 577 | 425(100), 407(61), 451(20) | EAF | [1] |
| 37 | (Epi)catechin-B-(epi)catechin hexoside *^c^* | 23.7 | 279 | 739 | 587(100)*^d^*, 435(100)*^e^*, 289(5) | ME, EAF, BF | [6] |
| 38 | 5-*O*-Feruloylquinic acid | 24.5 | 214, 323 | 367 | 191(100), 173(3) | DEF, EAF | [2] |
| 39 | Caffeic acid hexoside derivative *^c^* | 25.6 | 215, 325 | 425 | 341(100), 203(54), 179(34) | ME, DEF, EAF, BF | [1] |
| 40 | (Epi)catechin-B-(epi)catechin hexoside *^c^* | 26.3 | 279 | 739 | 577(100)*^d^*, 425(100)*^e^*, 407(31) | EAF | [6] |
| 41 | Kaempferol *O*-dihexoside (diHK) | 27.2 | 267, 340 | 609 | 447(6), 285(100) | ME, EAF, BF | [1] |
| 42 | (Epi)catechin-B-(epi)catechin hexoside *^c^* | 27.8 | 279 | 739 | 587(100)*^d^*, 407(42)*^e^*, 289(23) | EAF, BF | [6] |
| 43 | Kaempferol *O*-dihexoside | 28.0 | 266, 343 | 609 | 447(13), 429(55), 285(100) | ME, BF | [1] |
| 44 | (Epi)catechin-B-(epi)catechin hexoside *^c^* | 28.8 | 279 | 739 | 587(100), 451(18), 289(6) | EAF | [6] |
| 45 | (Epi)catechin-B-(epi)catechin hexoside | 29.1 | 279 | 739 | 587(100)*^d^*, 435(100)*^e^*, 289(18) | EAF | [6] |
| 46 | Quercetin *O*-hexosyl-pentoside | 29.2 | 269, 342 | 595 | 433(15), 415(41), 301(100) | ME, BF | [1] |
| 47 | Eriodictyol *O*-hexoside 1 (EH1) | 30.3 | 283, 330 | 449 | 287(100) | ME, DEF, EAF, BF | [7] |
| 48 | Eriodictyol *O*-hexoside 2 (EH2) *^c^* | 30.5 | 283, 330 | 449 | 287(100) | ME, DEF, EAF, BF | [7] |
| 49 | Quercetin 3-*O*-β-D-(6′′-*O*-α-L-rhamnosyl)-glucoside (RT, rutin) *^ab^* | 31.1 | 256, 353 | 609 | 447(100)*^d^*, 301(100)*^e^* | ME, EAF, BF | [8] |
| 50 | Quercetin 3-*O*-β-D-galactoside (HY, hyperoside)*^ab^* | 31.5 | 255, 354 | 463 | 301(100) | ME, DEF, EAF, BF | [8] |
| 51 | Quercetin 3-*O*-β-D-glucoside (IQ, isoquercitrin)*^ab^* | 32.9 | 256, 353 | 463 | 301(100) | ME, DEF, EAF, BF | [8] |
| 52 | Caffeic acid hexoside derivative *^c^* | 33.1 | 215, 325 | 425 | 341(100), 179(9) | ME, DEF, EAF, BF | [9] |
| 53 | Kaempferol *O*-hexoside | 33.3 | 264, 344 | 447 | 285(100) | ME, DEF, EAF, BF | [1] |
| 54 | (Epi)catechin derivative | 35.2 | 256 | 483 | 451(44), 341(22), 289(100) | ME, DEF, EAF |  |
| 55 | Cinchonain I isomer | 35.7 | 280 | 451 | 341(70), 299(100) | DEF | [9] |
| 56 | (Epi)catechin-B-(epi)catechin-B-(epi)catechin hexoside *^c^* | 36.1 | 279 | 1027 | 875(45)*^d^*, 857(100)*^e^*, 575(80)*^e^* | EAF | [6] |
| 57 | (Epi)catechin-B-(epi)catechin-B-(epi)catechin hexoside *^c^* | 36.5 | 279 | 1027 | 875(45)*^d^*, 857(100)*^e^*, 575(35)*^e^* | EAF | [6] |
| 58 | Kaempferol *O*-hexoside | 36.8 | 264, 344 | 447 | 285(100) | EAF | [1] |
| 59 | Kaempferol *O*-rhamnosyl-hexoside | 38.1 | 277, 335 | 593 | 447(28), 285(100) | ME, EAF, BF | [1] |
| 60 | Ferulic acid isomer (FAI) | 38.9 | 217, 321 | 193 |  | DEF | [1] |
| 61 | Caffeic acid derivative *^c^* | 39.2 | 215, 324 | 567 | 521(100), 345(10), 179(66) | ME, EAF, BF |  |
| 62 | Naringenin *O*-hexoside (NH) *^c^* | 40.0 | 283, 330 | 433 | 271(100) | ME, DEF, EAF, BF | [10] |
| 63 | 3,5-*O*-Dicaffeoylquinic acid (3,5-diCFQA) | 40.5 | 217, 326 | 515 | 353(100)*^d^*, 191(100)*^e^*, 179(30)*^e^* | ME, DEF, EAF | [11] |
| 64 | Cinchonain I isomer | 41.0 | 280 | 451 | 341(100), 299(15) | ME, DEF, EAF | [9] |
| 65 | Caffeic acid acetyl-hexoside derivative *^c^* | 41.8 | 215, 325 | 467 | 383(100), 179(54) | ME, DEF, EAF | [3] |
| 66 | 1,5-*O*-Dicaffeoylquinic acid (1,5-diCFQA) | 42.1 | 217, 326 | 515 | 353(100)*^d^*, 191(100)*^e^*, 179(41)*^e^* | ME, DEF, EAF | [11] |
| 67 | Caffeic acid acetyl-hexoside derivative *^c^* | 44.5 | 215, 325 | 467 | 383(68), 323(28), 179(100) | DEF, EAF | [3] |
| 68 | Caffeic acid acetyl-hexoside derivative *^c^* | 45.5 | 215, 325 | 467 | 383(100), 323(20), 179(99) | DEF, EAF | [3] |
| 69 | 4,5-*O*-Dicaffeoylquinic acid (4,5-diCFQA) | 46.1 | 218, 325 | 515 | 353(100)*^d^*, 179(60)*^e^*, 173(100)*^e^* | ME, DEF, EAF | [11] |
| 70 | Caffeic acid acetyl-hexoside derivative *^c^* | 47.1 | 215, 325 | 467 | 383(100)*^d^*, 323(30), 179(100)*^e^* | DEF, EAF | [3] |
| 71 | Cinchonain I isomer | 47.9 | 280 | 451 | 341(100) | DEF | [9] |
| 72 | Caffeic acid acetyl-hexoside derivative *^c^* | 48.0 | 215, 325 | 467 | 383(100), 323(12), 179(50) | ME, DEF, EAF | [3] |
| 73 | Cinchonain I isomer derivative *^c^* | 51.3 | 280 | 645 | 451(100), 341(49) | DEF | [9] |
| 74 | Caffeic acid derivative *^c^* | 51.5 | 215, 324 | 539 | 493(100), 375(60), 179(31) | ME, EAF |  |
| 75 | Cinchonain I isomer | 53.5 | 280 | 451 | 341(100) | ME, DEF, EAF | [9] |
| 76 | Cinchonain I isomer derivative *^c^* | 55.2 | 280 | 645 | 451(100)*^d^*, 341(100)*^e^* | DEF | [9] |
| 77 | Eriodictyol (ER) ^a^*^c^* | 55.7 | 283, 330 | 287 |  | DEF |  |
| 78 | Quercetin (QU) *^ab^* | 57.4 | 268, 364 | 301 |  | DEF |  |

*^a^* Compounds identified with authentic standards. *^b^* Compounds detected previously in *S. commixta* leaves. *^c^* Compounds detected for the first time in *Sorbus* species. *^d^* MS^2^ fragment ions subjected to MS^3^ fragmentation. *^e^* MS^3^ fragment ions. R_t_, time of retention; UV λ_max_, absorbance maxima in PDA spectra; [M−H]^−^, pseudomolecular ions in MS spectra recorded in a negative mode; ME, methanol-water (7:3, *v/v*) extract; DEF, diethyl ether fraction; EAF, ethyl acetate fraction; BF, *n*-butanol fraction; WR, water residue. Intensities of particular ions are given in parentheses. Nomenclature of the pseudodepsides of quinic acid and shikimic acid is given according to IUPAC.

**Supplementary Table S2.** Correlation coefficients (r) and probability (*p*) values of the linear relationships between phenolic contents in the *S. commixta* leaf extract/fractions and their activity parameters.

| r (p) | 3-NT | LOOHs | TBARS | NEAC | GUL | AMY | AGEs | TNF-α | IL-1β | IL-2 | IL-6 |
| --- | --- | --- | --- | --- | --- | --- | --- | --- | --- | --- | --- |
| PCA | 0.2513 | -0.7897 | 0.0088 | 0.3799 | -0.1546 | -0.2340 | -0.4098 | 0.5288 | -0.0364 | 0.1623 | 0.8574 |
|  | (0.683) | (0.112) | (0.989) | (0.528) | (0.804) | (0.705) | (0.493) | (0.360) | (0.954) | (0.794) | (0.063) |
| pHBA | 0.2513 | -0.7897 | 0.0088 | 0.3799 | -0.1546 | -0.2340 | -0.4098 | 0.5288 | -0.04 | 0.1623 | 0.8574 |
|  | (0.683) | (0.120) | (0.989) | (0.528) | (0.804) | (0.705) | (0.493) | (0.360) | (0.954) | (0.794) | (0.063) |
| FAI | 0.2513 | -0.7897 | 0.0088 | 0.3799 | 0.1546 | 0.2340 | 0.4098 | 0.5288 | 0.0364 | 0.1623 | 0.8574 |
|  | (0.683) | (0.112) | (0.989) | (0.528) | (0.804) | (0.705) | (0.493) | (0.360) | (0.954) | (0.794) | (0.063) |
| CFA | 0.2513 | -0.7897 | 0.0088 | 0.3799 | -0.1546 | -0.2340 | -0.4098 | 0.5288 | -0.0364 | 0.1623 | 0.8574 |
|  | (0.683) | (0.112) | (0.989) | (0.528) | (0.804) | (0.705) | (0.493) | (0.360) | (0.954) | (0.794) | (0.063) |
| NCHA | -0.3340 | 0.4721 | -0.5326 | -0.3283 | 0.7926 | 0.7982 | **0.8881** | -0.0533 | -0.0464 | 0.1078 | **-0.9008** |
|  | (0.583) | (0.422) | (0.355) | (0.590) | (0.110) | (0.105) | **(0.044)** | (0.932) | (0.941) | (0.863) | **(0.037)** |
| CHA | -0.0055 | 0.6135 | 0.0087 | 0.1449 | 0.6279 | 0.7238 | 0.7947 | 0.1203 | -0.0830 | 0.4586 | -0.7620 |
|  | (0.993) | (0.271) | (0.989) | (0.816) | (0.257) | (0.167) | (0.108) | (0.847) | (0.894) | (0.437) | (0.134) |
| CCHA | 0.4056 | -0.4608 | 0.4102 | 0.8078 | -0.2011 | -0.1773 | -0.3925 | 0.6775 | -0.2465 | 0.5097 | **0.8933** |
|  | (0.498) | (0.435) | (0.493) | (0.098) | (0.746) | (0.775) | (0.513) | (0.209) | (0.689) | (0.380) | **(0.041)** |
| cis-CHA | 0.3083 | 0.5411 | 0.5733 | 0.4759 | 0.1215 | 0.2691 | 0.3190 | 0.1282 | -0.0397 | 0.5263 | -0.3484 |
|  | (0.614) | (0.346) | (0.312) | (0.418) | (0.846) | (0.662) | (0.601) | (0.837) | (0.949) | (0.362) | (0.566) |
| 3,5-diCFQA | 0.6106 | -0.3988 | 0.7432 | 0.8116 | -0.4332 | -0.3784 | -0.5062 | 0.5024 | -0.0097 | 0.4703 | 0.8034 |
|  | (0.274) | (0.506) | (0.150) | (0.095) | (0.466) | (0.530) | (0.384) | (0.388) | (0.988) | (0.424) | (0.101) |
| 1,5-diCFQA | 0.6026 | -0.2440 | 0.8144 | 0.8217 | -0.4416 | -0.3602 | -0.4689 | 0.4414 | -0.0326 | 0.4890 | 0.7036 |
|  | (0.282) | (0.692) | (0.093) | (0.088) | (0.457) | (0.552) | (0.426) | (0.457) | (0.959) | (0.403) | (0.185) |
| 4,5-diCFQA | 0.6310 | -0.2361 | 0.8477 | 0.8134 | -0.4510 | -0.3669 | -0.4595 | 0.4199 | 0.0110 | 0.4881 | 0.6679 |
|  | (0.254) | (0.702) | (0.070) | (0.094) | (0.446) | (0.544) | (0.436) | (0.482) | (0.986) | (0.404) | (0.218) |
| 5-CFSA | 0.6131 | -0.4361 | 0.7250 | 0.8040 | -0.4300 | -0.3821 | -0.5123 | 0.5133 | 0.0015 | 0.4631 | 0.8212 |
|  | (0.271) | (0.463) | (0.166) | (0.101) | (0.470) | (0.526) | (0.378) | (0.376) | (0.998) | (0.432) | (0.088) |
| pCA | 0.2513 | -0.7897 | 0.0088 | 0.3799 | -0.1546 | -0.2340 | -0.4098 | 0.5288 | -0.0364 | 0.1623 | 0.8574 |
|  | (0.683) | (0.112) | (0.989) | (0.528) | (0.804) | (0.705) | (0.493) | (0.360) | (0.954) | (0.794) | (0.063) |
| 5-CQA | 0.4064 | 0.4324 | 0.6977 | 0.6052 | -0.0025 | 0.1479 | 0.1682 | 0.2043 | -0.0455 | 0.5707 | -0.1485 |
|  | (0.497) | (0.467) | (0.190) | (0.279) | (0.997) | (0.812) | (0.787) | (0.742) | (0.942) | (0.315) | (0.812) |
| SQ | -0.1300 | 0.2062 | -0.4892 | 0.0329 | **0.9794** | **0.9786** | **0.9813** | 0.3886 | -0.0965 | 0.4638 | -0.6610 |
|  | (0.835) | (0.739) | (0.403) | (0.958) | **(0.004)** | **(0.004)** | **(0.003)** | (0.518) | (0.877) | (0.431) | (0.224) |
| RT | -0.0279 | 0.2680 | -0.3247 | 0.1739 | **0.9511** | **0.9802** | **0.9830** | 0.4395 | -0.0999 | 0.5814 | -0.6533 |
|  | (0.965) | (0.663) | (0.594) | (0.780) | **(0.013)** | **(0.003)** | **(0.003)** | (0.459) | (0.873) | (0.304) | (0.232) |
| diHQ | -0.1244 | 0.1816 | 0.4981 | 0.0271 | **0.9823** | **0.9771** | **0.9802** | 0.3944 | -0.0831 | 0.4599 | -0.6545 |
|  | (0.842) | (0.770) | (0.393) | (0.966) | **(0.003)** | **(0.004)** | **(0.003)** | (0.511) | (0.894) | (0.436) | (0.231) |
| diHK | -0.1101 | 0.1699 | -0.4911 | 0.0152 | **0.9793** | **0.9722** | **0.9836** | 0.3849 | -0.0536 | 0.4533 | -0.6686 |
|  | (0.860) | (0.785) | (0.401) | (0.981) | **(0.004)** | **(0.006)** | **(0.003)** | (0.522) | (0.932) | (0.443) | (0.217) |
| HY | 0.4934 | 0.1173 | 0.8486 | 0.7482 | -0.3998 | -0.2723 | -0.3364 | 0.2679 | -0.1055 | 0.4700 | 0.4228 |
|  | (0.398) | (0.851) | (0.069) | (0.146) | (0.505) | (0.658) | (0.580) | (0.663) | (0.866) | (0.424) | (0.478) |
| IQ | 0.5198 | 0.0140 | 0.8393 | 0.7881 | -0.4149 | -0.2992 | -0.3852 | 0.3329 | -0.1089 | 0.4853 | 0.5283 |
|  | (0.369) | (0.982) | (0.075) | (0.113) | (0.487) | (0.625) | (0.522) | (0.584) | (0.862) | (0.407) | (0.360) |
| EH | 0.5947 | 0.0998 | 0.8532 | 0.8171 | -0.2030 | -0.0720 | -0.1151 | 0.3909 | -0.0171 | 0.6345 | 0.2734 |
|  | (0.290) | (0.873) | (0.066) | (0.091) | (0.743) | (0.908) | (0.854) | (0.515) | (0.978) | (0.250) | (0.656) |
| HN | 0.5922 | -0.0515 | **0.8860** | 0.7818 | -0.4379 | -0.3284 | -0.3935 | 0.3283 | -0.0106 | 0.4834 | 0.5191 |
|  | (0.293) | (0.934) | **(0.045)** | (0.118) | (0.461) | (0.590) | (0.512) | (0.590) | (0.986) | (0.409) | (0.370) |
| ER | 0.2513 | -0.7897 | 0.0088 | 0.3799 | -0.1546 | -0.2340 | -0.4098 | 0.5288 | -0.0364 | 0.1623 | 0.8574 |
|  | (0.683) | (0.112) | (0.989) | (0.528) | (0.804) | (0.705) | (0.493) | (0.360) | (0.954) | (0.794) | (0.063) |
| QU | 0.2513 | -0.7897 | 0.0088 | 0.3799 | -0.1546 | -0.2340 | -0.4098 | 0.5288 | -0.0364 | 0.1623 | 0.8574 |
|  | (0.683) | (0.112) | (0.989) | (0.528) | (0.804) | (0.705) | (0.493) | (0.360) | (0.954) | (0.794) | (0.063) |
| ECA | 0.4079 | 0.3339 | 0.8195 | 0.6188 | -0.3442 | -0.1985 | -0.2116 | 0.1121 | -0.0921 | 0.4101 | 0.1704 |
|  | (0.495) | (0.583) | (0.089) | (0.266) | (0.571) | (0.749) | (0.733) | (0.858) | (0.883) | (0.493) | (0.784) |
| CI | 0.6191 | -0.6390 | 0.6061 | 0.7260 | -0.3999 | -0.3933 | -0.5256 | 0.5494 | 0.0884 | 0.4028 | **0.8806** |
|  | (0.265) | (0.246) | (0.279) | (0.165) | (0.505) | (0.513) | (0.363) | (0.337) | (0.888) | (0.501) | **(0.049)** |
| TPhA | 0.6323 | -0.1624 | 0.7023 | **0.9793** | -0.0096 | 0.0877 | -0.0380 | 0.7125 | -0.0961 | 0.8131 | 0.4741 |
|  | (0.252) | (0.794) | (0.186) | **(0.004)** | (0.988) | (0.888) | (0.952) | (0.177) | (0.878) | (0.094) | (0.420) |
| TFL | 0.6117 | -0.0115 | 0.6163 | **0.9354** | 0.2599 | 0.3692 | 0.2864 | 0.7308 | -0.0545 | **0.9205** | 0.1510 |
|  | (0.273) | (0.985) | (0.268) | **(0.020)** | (0.673) | (0.541) | (0.640) | (0.161) | (0.931) | **(0.027)** | (0.809) |
| TPA | 0.0914 | 0.3790 | -0.0940 | 0.1531 | 0.8036 | 0.8617 | **0.9364** | 0.2806 | 0.0571 | 0.5460 | -0.7793 |
|  | (0.884) | (0.529) | (0.880) | (0.806) | (0.101) | (0.060) | **(0.019)** | (0.647) | (0.927) | (0.341) | (0.120) |
| TPH | 0.6336 | -0.1052 | 0.6790 | **0.9768** | 0.0965 | 0.2000 | 0.0894 | 0.7304 | -0.0813 | 0.8677 | 0.3539 |
|  | (0.251) | (0.866) | (0.207) | **(0.004)** | (0.877) | (0.747) | (0.886) | (0.161) | (0.897) | (0.057) | (0.559) |
| TPC | 0.6343 | -0.4657 | 0.4907 | **0.9659** | 0.1326 | 0.1736 | 0.0068 | **0.8947** | -0.0616 | 0.8354 | 0.6053 |
|  | (0.250) | (0.429) | (0.401) | **(0.008)** | (0.832) | (0.780) | (0.991) | **(0.040)** | (0.922) | (0.078) | (0.279) |

Activity and concentration parameters according to Table 1 and Figures 3-5. The bold mean statistical significance of the estimated linear relationships (*p* < 0.05). For statistical analysis the activity in human plasma model and cellular studies were calculated as percentage of decrease (for 3-NT, LOOHs, TBARS, IL-1β, IL-2, IL-6, and TNF-α) or increase (for NEAC) of the tested parameter in the plasma treated with ONOO^−^ in the presence of the investigated extracts versus ONOO^−^ -treated plasma in the absence of the analytes. Abbreviations: PCA, protocatechuic acid; pHBA, *p*-hydroxybenzoic acid; FAI, ferulic acid isomer; CFA, caffeic acid, NCHA, neochlorogenic acid; CHA, chlorogenic acid; CCHA, cryptochlorogenic acid; cis-CHA, cis-chlorogenic acid; 3,5-diCFQA, 3,5-*O*-dicaffeoylquinic acid; 1,5-diCFQA, 1,5-*O*-dicaffeoylquinic acid; 4,5-diCFQA, 4,5-*O*-dicaffeoylquinic acid; 5-CFSA, 5-*O*-caffeoylshikimic acid; pCA, *p*-coumaric acid; 5-CQA, 5*-O-p*-coumaroylquinic acid; SQ, quercetin 3-*O*-β-sophoroside; RT, rutin; diHQ, quercetin *O*-dihexoside; diHK, kaempferol *O*-dihexoside; HY, hyperoside; IQ, isoquercitrin; EH, eriodictyol *O*-hexosides (two compounds) 1; NH, naringenin *O*-hexoside; ER, eriodictyol; QU, quercetin; EC; (-)-epicatechin; CI, cinchonain I isomers; TPhA, total phenolic acids (determined by HPLC-PDA); TFL, total flavonoids (determined by HPLC-PDA); TPA, total proanthocyanidins (determined by *n*-butanol/HCl assay); TPH, total polyphenols (determined by HPLC-PDA); TPC, total polyphenols (determined by Folin-Ciocalteu assay); 3-NT, effects on the nitration of tyrosine residues; LOOHs, effects on the plasma lipids oxidation and the formation of hydroperoxides; TBARS, effects on the plasma lipids oxidation and the formation of thiobarbituric acid reactive substances; NEAC, effects on the non-enzymatic antioxidant capacity of plasma; GLU, α-glucosidase inhibition; AMY, α-amylase inhibition; AGEs, inhibition of the advanced glycation end products formation; IL-1β, IL-2, IL-6, TNF-α, inhibition of cytokines release.

**Supplementary Figure S1.** Viability of peripheral blood mononuclear cells (PBMCs) after 24 h of incubation with *S. commixta* leaf extract/fractions at 1-50 µg/mL tested using resazurin as a spectrophotometric dye. Results expressed as means ± SD (*n* = 12) and presented as relative percentage (assuming the cells viability in control samples (without analytes) as 100%). All values are not statistically different (*p* > 0.05).

**References**

1.	Olszewska, M. A. *et al.* The Effect of Standardised Flower Extracts of *Sorbus aucuparia* L. on Proinflammatory Enzymes, Multiple Oxidants, and Oxidative/Nitrative Damage of Human Plasma Components In Vitro. *Oxid. Med. Cell. Longev.* **2019**, 1–18. doi:10.1155/2019/9746358 (2019)

2.	Clifford, M. N., Johnston, K. L., Knight, S. & Kuhnert, N. Hierarchical Scheme for LC-MS n Identification of Chlorogenic Acids. *J. Agric. Food Chem.* **51**, 2900–2911. doi:10.1021/jf026187q (2003).

3.	Barreira, J. C. M. *et al.* Phenolic profiling of *Veronica* spp. grown in mountain, urban and sandy soil environments. *Food Chem.* **163**, 275–283. doi:10.1016/j.foodchem.2014.04.117 (2014).

4.	Gonçalves, F. M. B., Sousa, A. L. D. & Oliveira, R. R. D. Use of mass spectrometry as a tool for the search or identification of flavonoids in Urticaceae. *Rodriguésia* **74**, e01152022. doi:10.1590/2175-7860202374049 (2023).

5.	Clifford, M. N., Kirkpatrick, J., Kuhnert, N., Roozendaal, H. & Salgado, P. R. LC–MSn analysis of the cis isomers of chlorogenic acids. *Food Chem.* **106**, 379–385. doi:10.1016/j.foodchem.2007.05.081 (2008).

6.	Zerbib, M., Cazals, G., Enjalbal, C. & Saucier, C. Identification and Quantification of Flavanol Glycosides in *Vitis vinifera* Grape Seeds and Skins during Ripening. *Molecules* **23**, 2745. doi:10.3390/molecules23112745 (2018).

7.	Mikulic-Petkovsek, M., Krska, B., Kiprovski, B. & Veberic, R. Bioactive Components and Antioxidant Capacity of Fruits from Nine *Sorbus* Genotypes. *J. Food Sci.* **82**, 647–658. doi:10.1111/1750-3841.13643 (2017).

8.	Olszewska, M. A., Presler, A. & Michel, P. Profiling of Phenolic Compounds and Antioxidant Activity of Dry Extracts from the Selected *Sorbus* Species. *Molecules* **17**, 3093–3113. doi:10.3390/molecules17033093 (2012).

9.	Rutkowska, M., Balcerczak, E., Świechowski, R., Dubicka, M. & Olszewska, M. A. Seasonal variation in phenylpropanoid biosynthesis and in vitro antioxidant activity of *Sorbus domestica* leaves: Harvesting time optimisation for medicinal application. *Ind. Crop. Prod.* **156**, 112858. doi:10.1016/j.indcrop.2020.112858 (2020).

10.	Bystrom, L. M., Lewis, B. A., Brown, D. L., Rodriguez, E. & Obendorf, R. L. Characterisation of phenolics by LC–UV/Vis, LC–MS/MS and sugars by GC in *Melicoccus bijugatus* Jacq. ‘Montgomery’ fruits. *Food Chem.* **111**, 1017–1024. doi:10.1016/j.foodchem.2008.04.058 (2008).

11.	Clifford, M. N., Knight, S. & Kuhnert, N. Discriminating between the Six Isomers of Dicaffeoylquinic Acid by LC-MS(n). *J. Agric. Food Chem.* **53**, 3821–3832. doi:10.1021/jf050046h (2005).
